# Transcriptome Profiling of the Cancer, Adjacent Non-Tumor and Distant Normal Tissues from a Colorectal Cancer Patient by Deep Sequencing

**DOI:** 10.1371/journal.pone.0041001

**Published:** 2012-08-08

**Authors:** Yan'an Wu, Xuetao Wang, Fangbo Wu, Ruolei Huang, Fangqin Xue, Guantao Liang, Min Tao, Pengwei Cai, Yi Huang

**Affiliations:** 1 Department of Clinical Laboratory, Fujian Provincial Hospital, Fujian Provincial Clinical Medical College, Fujian Medical University, Fuzhou, China; 2 Department of Tumor Surgery, Fujian Provincial Hospital, Fujian Provincial Clinical Medical College, Fujian Medical University, Fuzhou, China; University of Bari & Consorzio Mario Negri Sud, Italy

## Abstract

Colorectal cancer (CRC) is one of the most commonly diagnosed cancers in the world. A genome-wide screening of transcriptome dysregulation between cancer and normal tissue would provide insight into the molecular basis of CRC initiation and progression. Compared with microarray technology, which is commonly used to identify transcriptional changes, the recently developed RNA-seq technique has the ability to detect other abnormal regulations in the cancer transcriptome, such as alternative splicing, novel transcripts or gene fusion. In this study, we performed high-throughput transcriptome sequencing at ∼50× coverage on CRC, adjacent non-tumor and distant normal tissue. The results revealed cancer-specific, differentially expressed genes and differential alternative splicing, suggesting that the extracellular matrix and metabolic pathways are activated and the genes related to cell homeostasis are suppressed in CRC. In addition, one tumor-restricted gene fusion, PRTEN-NOTCH2, was also detected and experimentally confirmed. This study reveals some common features in tumor invasion and provides a comprehensive survey of the CRC transcriptome, which provides better insight into the complexity of regulatory changes during tumorigenesis.

## Introduction

Colorectal cancer (CRC) is one of the most commonly diagnosed cancers with over one million new cases worldwide in every year [1]. Metastatic CRC is usually incurable; as a result, CRC is the leading cause of cancer-related deaths [1], [2]. CRC arises from adenomatous polyps and develops into locally invasive and subsequently metastatic cancer. The progression of CRC is a multistep process and can be categorized into four stages (Dukes staging system) based on the degree of tumor invasion [3], [4]. In previous studies, several molecular mechanisms, such as genomic instability [5], [6], [7], loss of DNA repair genes [8], [9] and aberrant epigenetic modifications [10], [11] (see review in [12]), were shown to contribute to the development of CRC. Additionally, an unbiased approach of high-throughput screening of the expression changes between CRC and normal tissue revealed multiple diagnostic and prognostic biomarkers [13], [14], [15]. However, the comprehensive understanding of the progression of CRC and the proper prognosis are still challenging task due to the genetic heterogeneity of CRC and complex genomic alterations found with this type of cancer [12], [16].

Prior studies of genomic alterations have revealed that somatic changes, including point mutations, DNA rearrangements and copy number variations (reviewed in [12]), can result in mutations that drive the development of CRC. As a consequence of changes in the cancer genome, the reprogramming of the transcriptome leads to abnormal cellular behavior and thus directly contributes to cancer progression [17], [18]. Studying the cancer transcriptome not only enables us to fill in the gap between driver mutations and cancer cell behavior, but also allows us to identify additional candidate cancer-related mutations and the molecular basis of gene regulation [17]. The recent development of massively parallel sequencing (RNA-seq) provides a powerful approach to profile the transcriptome with greater efficiency and higher resolution [19]. The advantage of RNA-seq is that this technique makes feasible the study of the cancer transcriptome complexity, including alternative splicing, isoform usage, gene fusions and novel transcripts (reviewed in [20], [21]). Despite the prevalence of using RNA-seq to study various cancer transcriptomes [22], [23], [24], [25], the deep annotation of CRC gene expression profiling has not been performed.

In this study, we aimed to thoroughly annotate the transcriptomes of CRC tissue, adjacent non-tumor tissue and distant normal tissue from a single patient by RNA-seq. First, we found several cancer-specific dysregulated genes and alternative splicing. Second, following Gene Ontology (GO) and pathway analysis of the dysregulated genes and isoforms, we identified a potential candidate pathway and a functional class of genes that are relevant to CRC progression, which has not been reported previously. Third, we detected a novel gene fusion event specifically in CRC tissue and experimentally confirmed the fusion product. Finally, to validate our sequencing results, quantitative real-time PCR (qPCR) was used to confirm the gene expression difference between CRC and normal tissue.

## Results

### Characterization of sequencing and mapping

Three samples – CRC tissue (stage III), adjacent non-tumor tissue and distant normal tissue – were collected from a 57-year-old female patient. The clinicopathological information of the patient is shown in Fig. S1. All three samples were subjected to massively parallel paired-end cDNA sequencing. In total, we obtained 36.5 million, 33.1 million and 29.9 million read pairs from the CRC, adjacent non-tumor and distant normal tissue, respectively. We used TopHat to align the reads to the UCSC (the University of California Santa) reference human genome Hg19. The uniquely aligned reads for the three samples ranged from 20.8 million to 25.9 million pairs. The proportion of reads that mapped to the Ensembl reference genes ranged from 75% to 86% for the three samples. The average coverage of our sequencing depth was approximately 50 times of human transcriptome (approximately 113 millon bp, based on the total length of the uniquely annotated exon region in the Ensembl database). In addition, only ∼1% reads were mapped to rRNA, indicating that our libraries are properly constructed and faithfully represent the expression of RNA with ployA tails. The details of the mapping results are listed in Table 1.

**Table 1:** Statistics of colorectal cancer transcriptome mapping to human genome Hg19.

Colorectal cancer transcriptome			
	Normal	Adjacent non-tumor	Cancer
**Total reads**	59,761,418 (100%)	66,100,224 (100%)	73,010,454 (100%)
**Uniquely Mapped Single Reads**	4,318,631 (7.2%)	5,092,024 (7.7%)	5,093,074 (7.0%)
**Uniquely Mapped Paired Reads**	41,569,814 (69.6%)	44,337,404 (67.1%)	51,803,606 (71.0%)
**Total Uniquely Mapped Reads**	45,888,445 (76.8%)	49,429,428 (74.8%)	56,896,680 (78.0%)
**Uniquely Splice Junction Reads**	6,833,565 (11.4%)	8,552,384 (13.0%)	7,014,337 (9.6%)
**Total Uniquely Mapped length** (**bp**)	5,277,776,724	5,451,838,964	6,198,952,281

### Analysis of differentially expressed genes

**Figure 1:**
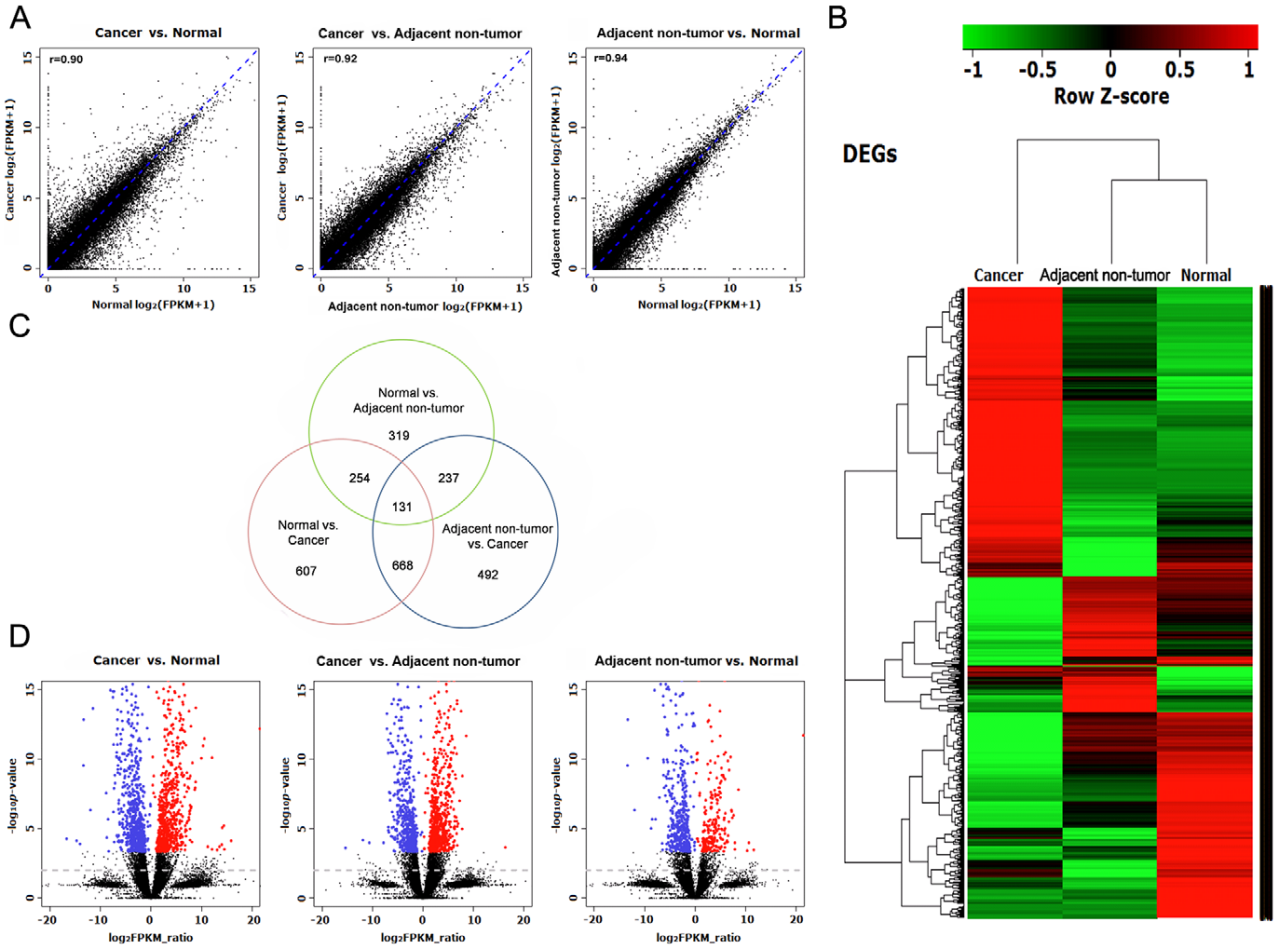
Differential expression analysis of cancer, adjacent non-tumor and distant normal tissue. A: The scatter plot for global expression between samples; the Pearson correlation coefficient is shown; B: Hierarchical clustering of differentially expressed genes (DEGs) among samples; C: Venn diagram to illustrate the overlapped DEGs between samples; D: Volcano plots for all the genes in each comparison. The red and blue dots indicate that up- and down-regulated DEGs were significant at q values less than 0.01.

To measure the gene expression and to identify the differentially expressed genes (DEGs) among the samples, we used the method of Cuffdiff [26] to estimate the gene expression and to identify significantly dysregulated genes. The normalized expression level of each gene was measured by Fragments Per Kilobase of exon per Million fragments mapped (FPKM). By requiring that the FPKM was greater than one, we detected 14854–15168 expressed genes in each sample, which included the majority of the annotated human reference genes (See Table S1 for details). We further analyzed the correlation of the gene expression among the samples. The global profiles of gene expression were generally highly correlated with the Pearson correlation coefficient, ranging from 0.90 to 0.94 (Fig. 1A). In addition, the clustering analysis indicates that the CRC transcriptome is distinguished from those of the adjacent non-tumor tissue and distant normal tissue (Fig. 1B).

We detected 1660, 1528 and 941 significant DEGs between the CRC and adjacent tissue, the CRC and normal tissue and the adjacent and normal tissue, respectively (the complete lists of the DEGs are summarized in Table S1). The overlapping of the DEGs among the three samples is shown as a Venn diagram in Fig. 1C. It is noteworthy that CRC yields more dysregulated genes (1660 genes in CRC vs. adjacent, 1528 genes in CRC vs. normal) than other two tissues, which are 1.5-fold more abundant than those found in other tissues (941 genes in normal vs. adjacent), indicating the cancer-specific reprogramming of the CRC transcriptome, as shown in the “volcano plot” of the gene expression profiles (Fig. 1D). When comparing the direction of the DEGs, the number of up- and down-regulated genes identified between CRC and the other two samples was nearly equal. In contrast, a slight increase in the down-regulated genes was observed in adjacent non-tumor tissue when compared to the adjacent cancer and normal tissue. The MA-plot of the gene expression profiles (Fig. S2) shows that the significant number of dysregulated genes is not biased toward highly expressed genes.

In previous studies, several key genes relevant to CRC have been identified. To determine whether our findings were in agreement with reported results, we systematically compared the changes in the expression of specific CRC-related genes with those identified in other studies. We found that 15-prostaglandin dehydrogenase (15-PGDH), a rate-limiting enzyme that catalyzes the degradation of prostaglandin [27], is significantly down-regulated in CRC in cancer tissues compared to normal tissues. The activation of COX-2 and the loss of 15-PGDH are common oncogenic events that are observed in ∼80% of CRC cases [28]. In addition, we found another tumor suppressor, *TGFBR2* [29], which was down-regulated in both CRC and cancer-adjacent tissues. Because the inactivation of TGFBR2 is coordinated with the transition from adenoma to carcinoma, the progressive inactivation of TGFBR2 in cancer-adjacent and tumor tissues is expected. We also detected other genes that were dysregulated in CRC, including APC [30], MYH [31], CD133, IDH1 and MINT2 [10]. In contrast, several known driver factors that are frequently mutated in CRC, including MINT3 [32], [33], MSH2 [34] and MSH6 [9], showed no change in expression in this study, suggesting that the genetic heterogeneity of CRC or the mutated products might be deleterious even if the expression level is unaffected.

### Functional enrichment analysis of differentially expressed genes

**Figure 2:**
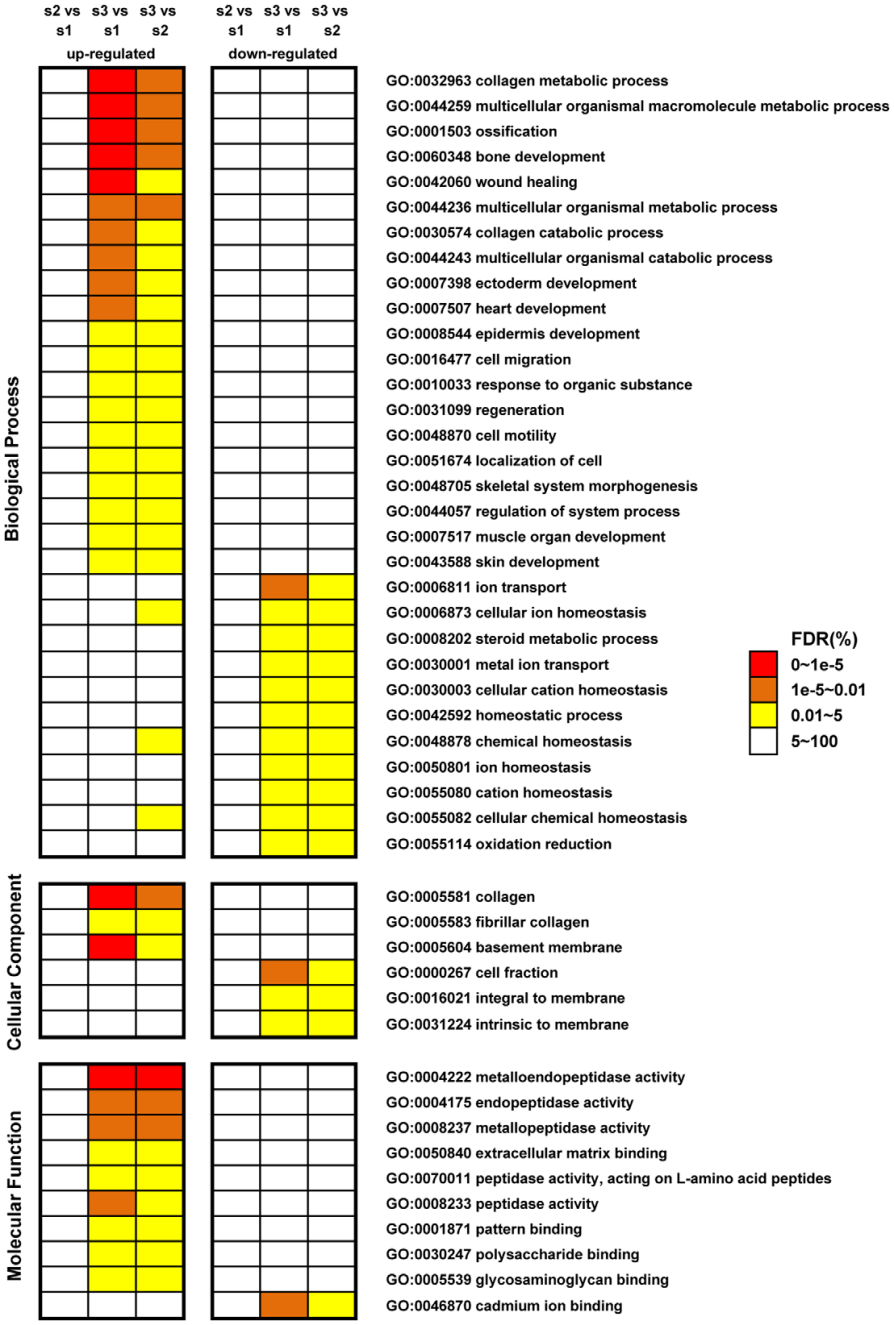
The functional enrichment of cancer-specific dysregulated genes identified in the Gene Ontology analysis. We only chose GO categories that enriched cancer-related dysregulated genes but did not enrich dysregulated genes that were identified when comparing normal tissue with adjacent non-tumor tissue. The cancer-specific dysregulated genes were categorized as significantly up- or down-regulated genes in cancer tissue. The level of significance is indicated by different colors. The “s1”, “s2” and “s3” indicators denote “cancer”, “adjacent non-tumor” and “distant normal” tissues, respectively.

To better understand the function of DEGs, we conducted an enrichment analysis of Gene Ontology for the dysregulated genes. To identify the cancer-specific functional categories, we first performed parallel enrichment tests for significantly up- and down-regulated genes that were detected by pair-wised comparisons in the CRC, adjacent non-tumor and normal tissue using online tools from DAVID [35]. The GO categories that were significantly enriched in the dysregulated genes from the comparison of CRC vs. cancer adjacent non-tumor tissue and CRC vs. normal tissue, but not adjacent non-tumor vs. normal tissue, were selected. In total, the up- and down-regulated genes in CRC were categorized into 47 cancer-specific functional categories (Fig. 2). Interestingly, although we identified equal numbers of up- and down-regulated genes in CRC, we observed an excess of significant GO categories for CRC up-regulated genes, suggesting that the up-regulation of cancer-specific genes is functionally more important for cancer progression. For example, the significant GO terms for up-regulated genes, which include “cell migration”, “cell motility” and “extracellular matrix binding”, are relevant to cancer invasion [15], [36], [37]. In addition, the genes related to metabolic changes, including “collagen metabolic process”, “multicellular organismal macromolecule metabolic process” and “multicellular organismal catabolic process”, reflect the alteration of tumor metabolism [38], [39] and are also over-represented in CRC. On the contrary, genes that are down-regulated in CRC are enriched in several functional processes related to homeostasis.

A more informative analysis of functional annotation can be achieved by studying the enrichment of differentially expressed genes in a particular pathway. We used DAVID [35] to analyze which KEGG pathway was enriched with CRC-specific dysregulated genes. The pathways enriched with DEGs are listed in Table 2. The extracellular matrix (ECM) receptor interaction pathway was commonly affected in all the pair-wise comparisons, and such gene regulation alterations in the ECM pathway were much more severe in the CRC tissue. In addition, the focal adhesion pathway was enriched in the DEGs identified from the CRC tissue.

**Table 2:** KEGG pathway of enriched differentially expressed genes.

Comparison	Pathway ID	Pathway Name	Fold enrichment	FDR (%)
**Cancer vs. Normal**	hsa04512	ECM-receptor interaction	3.57	1.6E-05
	hsa04510	Focal adhesion	1.86	1.1
	hsa04610	Complement and coagulation cascades	2.61	3.5
**Cancer vs. Adjacent non-tumor**	hsa04512	ECM-receptor interaction	3.9	8.0E-05
	hsa04510	Focal adhesion	1.93	2.3
	hsa05410	Hypertrophic cardiomyopathy (HCM)	2.76	1.5
	hsa05414	Dilated cardiomyopathy	2.61	2.5
**Adjacent non-tumor vs. Normal**	hsa04512	ECM-receptor interaction	3.8	2.5
	hsa04742	Taste transduction	6.24	2.6

**Figure 3:**
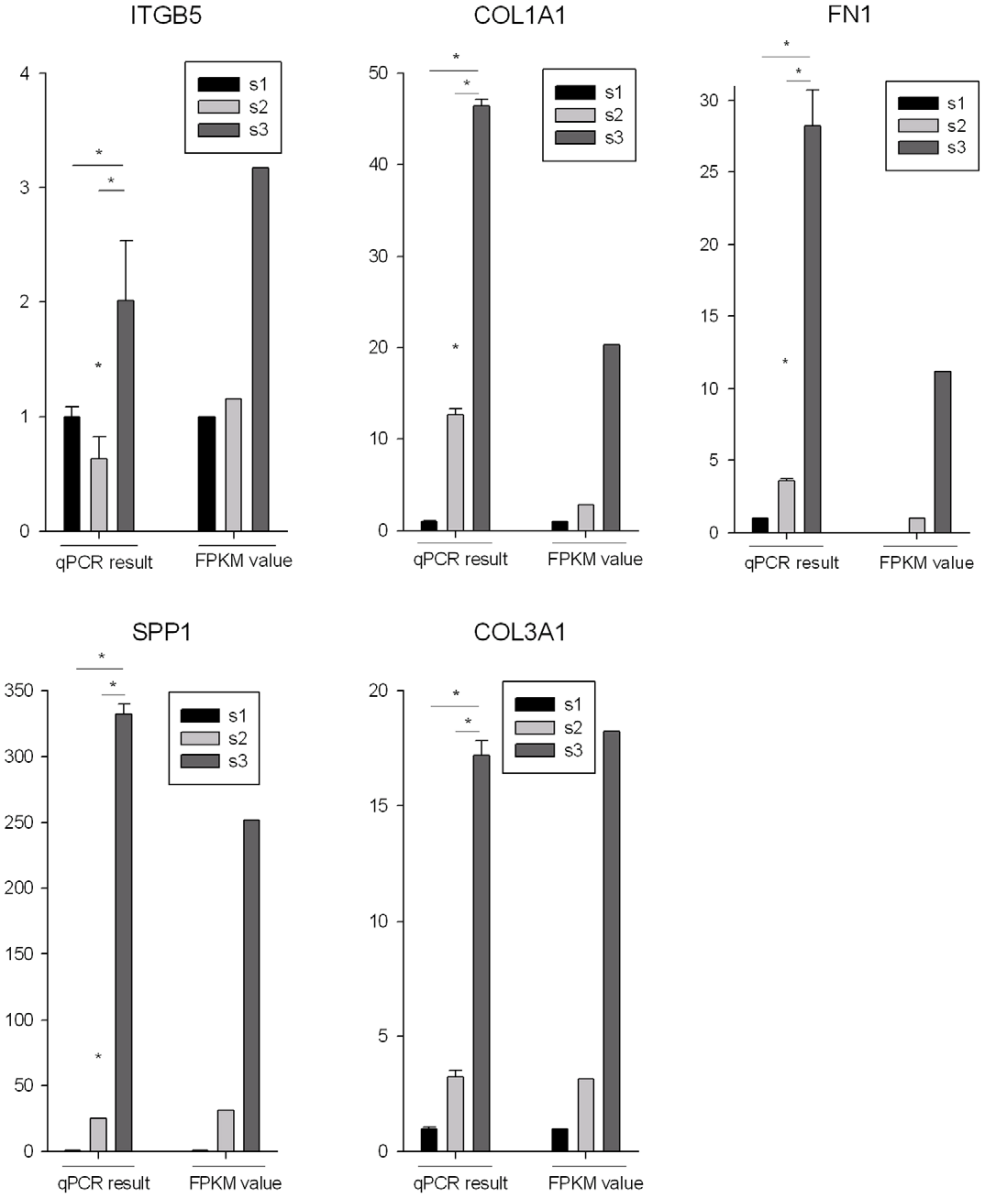
The differentially expressed genes detected by RNA-seq are confirmed by qRT-PCR. qRT-PCR was performed for five genes that are identified as differential expressed genes between CRC and other two tissues. The expression level of each gene was normalized to the level in normal tissue. The “s1”, “s2” and “s3” indicators denote “cancer”, “adjacent non-tumor” and “distant normal” tissues, respectively.

To experimentally confirm the differentially expressed genes identified by RNA-seq, the expression levels of selected genes were validated in each sample by quantitative real-time PCR (qRT-PCR). We chose five candidate genes (COL1A1, COL3A1, FN1, SPP1, and ITGB5) from the ECM pathway (according to the gene expression level and fold change between the CRC and normal tissue) that were differentially expressed by Cuffdiff (Table S2). We used GAPDH as an endogenous control in these reactions. The qRT-PCR results confirmed that all of these candidate genes showed nearly identical changes in gene expression to those detected via the RNA-seq technique, as shown in Fig. 3.

To examine whether these genes were always up-regulated in the colorectal cancer, we performed the qRT-PCR to test the expression changes for the five genes between the paired cancer and normal tissue in ten additional patients. The result of one paired samples from one patient was excluded due to large variation within technical replicates. The result of the remained patients showed that, except for ITGB5, the other three genes COL1A1, FN1and SPP1were up-regulated in six cancer samples, and the COL3A1 was up-regulated in four cancer samples (Table S3), suggesting the ECM-pathway genes are usually up-regulated in colorectal cancer. In addition, the cancer samples of five patients can be clustered together according to the expression levels of these five genes (Fig. S3).

### Analysis of alternative splicing and differential usage of isoforms

**Figure 4:**
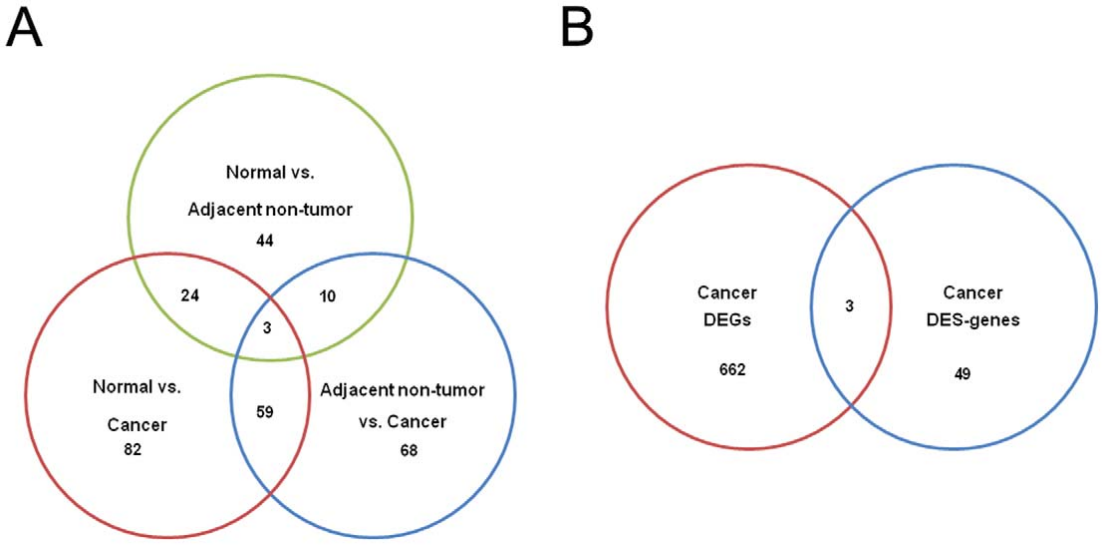
Analysis of differential exon skipping (**DES**) **events among samples.** A: Venn diagram of the number of DES events; B: The overlap between differentially expressed genes and genes with DES events.

One gene locus can express multiple isoforms by alternative splicing (AS). The transcript diversity leads to plastic transcriptional networks in cancer, which are important to generate the unusual properties of cancer cells [40], [41]. Of the numerous molecular mechanisms that can generate AS isoforms, exon skipping to truncate the functional domain is the most common way to generate protein products with alternative functions in mammals [41]. We thus performed genome-wide screening to identify the cancer-restricted exon skipping events using software MISO (the Mixture of Isoforms) [42]. In total, we detected 14072, 14537 and 13865 exon skipping events in the CRC, adjacent non-tumor tissue and normal tissue, respectively. We next compared the differential exon skipping (DES) events (Table S4) among samples, as shown in Fig. 4A. We found that: i) only a small proportion of DES events was shared in three way comparison, suggesting that a considerable proportion of genes are under cancer-specific regulation by alternative splicing; and ii) the number of DESs between normal and adjacent tissue is less than the number of DES events between the CRC and adjacent tissue or the CRC and normal tissue, indicating the enhanced use of differential isoforms in cancer. Because ES events are likely to change protein function by affecting the functional domain, the number of DES events in CRC tissue is roughly twice that of non-cancer tissue, indicating that the splicing pathway can be significantly activated in CRC to generate diverse functional products. We checked the expression of the slicing factors derived from NCBI and SpliceAid 2 (http://www.introni.it/spliceaid.html), and found that four splicing factors, including RBFOX1, SPRK1, MBNL1 and SRRM2, were significantly dysregulated between the cancer tissue and non-cancer tissue (Table S1), suggesting that the anomalous splicing activity in cancer tissue might be related with the dysregulation of the splicing factors.

While the DEGs have already been shown to be significant in CRC, it would be interesting to determine the degree of overlap between the DEGs and genes with DES events. As shown in Fig. 4B, only three genes (3/752, ∼0.4%) were simultaneously affected by changes in transcriptional regulation and post-transcriptional regulation (i.e., alternative splicing).

**Figure 5:**
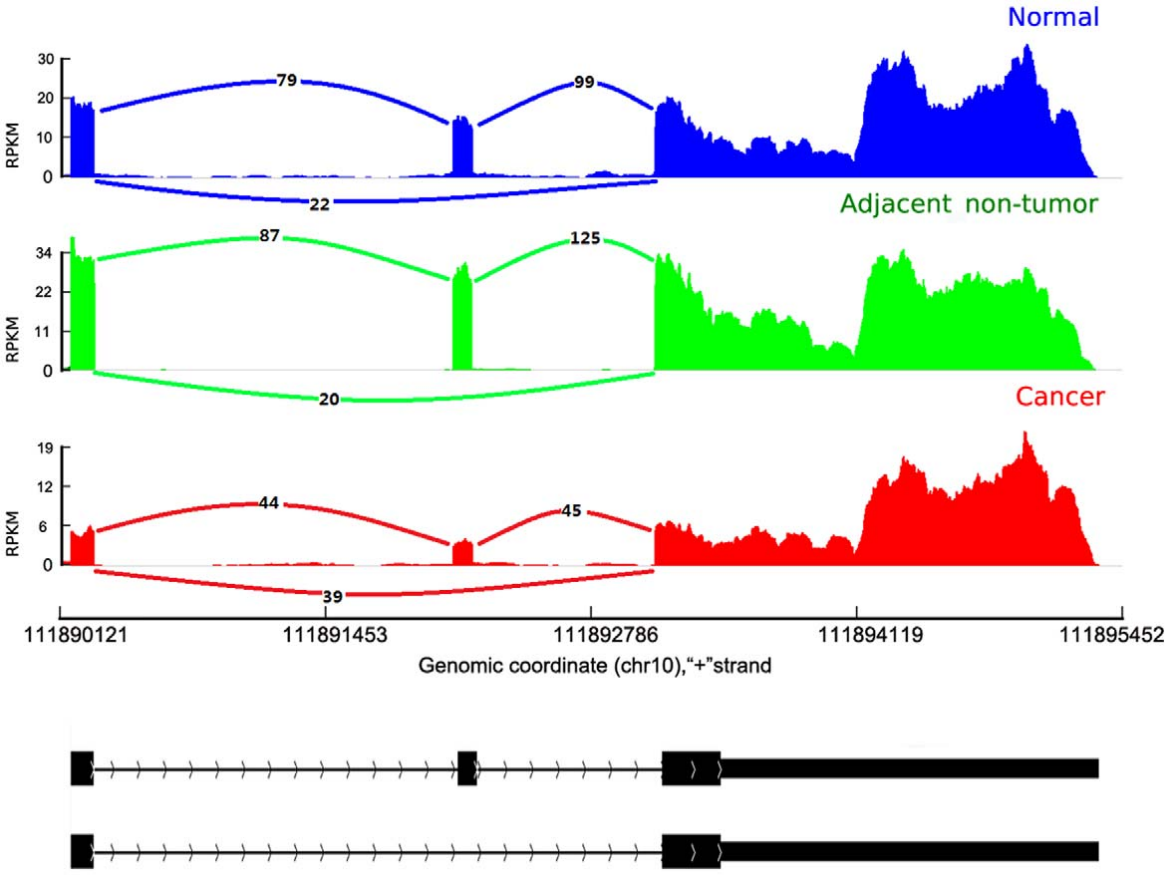
RNA-seq reads coverage of the gene ADD3. The RNA-Seq reads were mapping to the UCSC reference genome (hg19) of ADD3. The CRC tissue tracks were shown in red, the adjacent non-tumor in green and the normal tissue in blue. The counts of reads spanning the junction of exons were shown.

To identify highly reliable cancer-associated genes with DES events, we filtered the DES events by a series steps (Material and Methods) from all DES events (Table S4) and obtained 20 reliable DES events from 14 cancer-associated genes (Table 3). Six genes, including ADD3, CTNND1, EPB41L3, F3, MUC4 and PDGFA, showed cancer-tissue-specific DES events. As shown in Fig. 5. The ratio of junction-reads number for the exon inclusion versus the exon exclusion was obviously lower in the cancer tissue than that in the other two tissues. The reads mapping of other five genes were shown in Fig. S4–S8 respectively.

**Table 3:** Cancer-associated differential splicing events in colorectal cancer.

gene symbol	gene description	location of skipped exon	sample1&	sample2&	Ψ sample1#	Ψ sample2#	diff*	bayes factor$
ADD3	adducin 3 (gamma)	chr10:111892063–111892158	s1	s3	0.81	0.46	0.35	4.00E+27
			s2	s3	0.87	0.46	0.41	1.90E+47
CTNND1	catenin (cadherin-associated protein), delta 1	chr11:57583387–57583473	s1	s3	0.78	0.26	0.52	1.80E+202
			s2	s3	0.73	0.26	0.47	4.70E+184
EPB41L3	erythrocyte membrane protein band 4.1-like 3	chr18:5394676–5394792	s1	s3	0.3	0.76	−0.46	2.20E+19
			s2	s3	0.4	0.76	−0.36	1.10E+05
F3	coagulation factor III (thromboplastin, tissue factor)	chr1:95006128–95006622	s1	s3	0.27	0.9	−0.63	7.70E+73
			s2	s3	0.54	0.9	−0.36	1.10E+07
LMO7	LIM domain 7	chr13:76381616–76382335	s2	s3	0.01	0.33	−0.32	5.20E+06
MUC4	mucin 4, cell surface associated	chr3:195481084–195481243	s1	s3	0.89	0.57	0.32	1.20E+29
			s2	s3	0.89	0.57	0.32	5.00E+05
MYH11	myosin, heavy chain 11, smooth muscle	chr16:15802660–15802698	s1	s3	0.56	0.24	0.32	1.10E+284
PDGFA	platelet-derived growth factor alpha polypeptide	chr7:540068–540136	s1	s3	0.73	0.13	0.6	8.70E+101
			s2	s3	0.5	0.13	0.37	7.00E+09
PML	promyelocytic leukemia	chr15:74324913-74325056	s1	s3	0.65	0.31	0.34	1.20E+04
PRDM2	PR domain containing 2, with ZNF domain	chr1:14104913–14109326	s2	s3	0.84	0.4	0.44	4.30E+50
RAB40B	RAB40B, member RAS oncogene family	chr17:80616367–80616589	s2	s3	0.93	0.45	0.48	8.90E+07
TCF7L2	transcription factor 7-like 2 (T-cell specific, HMG-box)	chr10:114920378–114920450	s1	s3	0.9	0.33	0.57	1.10E+05
TSSC4	tumor suppressing subtransferable candidate 4	chr11:2423069–2423377	s1	s3	0.07	0.56	−0.49	7.80E+05
TTL	tubulin tyrosine ligase	chr2:113277859–113278002	s2	s3	0.84	0.46	0.38	1.00E+04

& : s1, s2 and s3 represented the normal, adjacent non-tumor and cancer tissues, respectively;

# : Ψ, percentage spliced in, denotes the fraction of mRNAs that represent the inclusion isoform [67];

* : The “diff” is provided by the MISO, and indicated the degree of splicing difference between samples. It was in [−1, 1]. The positive “diff” value means that the exon was skipped less in the sample1 than that in the sample2, and the negative values means the exon skipped less in the sample2;

$ : The “bayes factor” is provided by MISO, indicating the significance of the splicing difference. It was in [0, +∞), and it was greater, then the difference was more significant.

The differential splicing of ADD3 has been found in the non-small cell lung cancer [43] and the murine breast tumor [44]. Interestingly, ADD3 showed a cassette exon inclusion in these two studies, but it showed a cassette exon exclusion in the same location in our study. In another study of human embryonic stem cells (hESCs), the cassette exon exclusion has also been found in hESCs relative to the derived cardiac progenitors [45]. Even in the previous lung cancer study, there was also heterogeneous evidence for alternative splicing patterns of ADD3 (four of 18 lung-cancer patients showed a cassette exon exclusion for ADD3) [43]. Therefore, further studies on ADD3 should be done to understand the relationship of its alternative splicing with cancer.

### Bioinformatics prediction of gene fusion events

**Figure 6:**
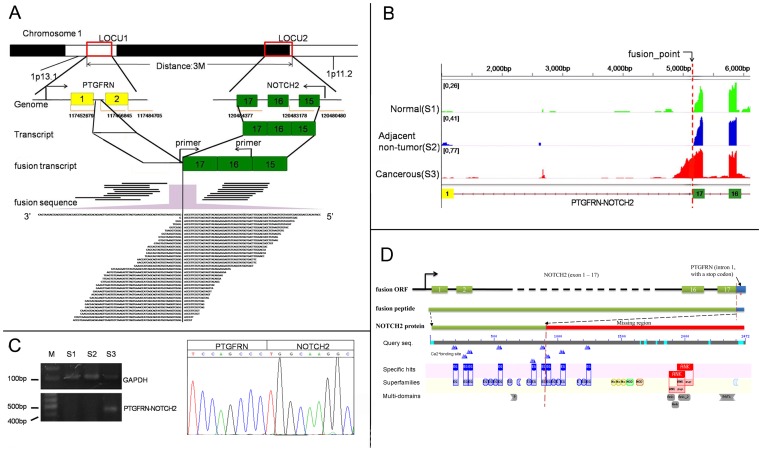
Illustration of the PTGFRN-NOTCH2 gene fusion in cancer tissue. A: The inter-chromosomal gene fusion involves the PTGFRN (shown in yellow) and NOTCH2 (shown in green) loci; the distance between these two loci is about three Mbp. The fusion events were detected by pair-end reads that spanned the fusion region and reads that crossed the fusion region; B: The comparison of the reads mapping results for the fusion transcripts among the three samples. The structure of the fusion gene is at the bottom. The reads counts for “normal”, “adjacent non-tumor” and “cancer” tissue are denoted as “green”, “blue” and “red” bars, respectively. C: The RT-PCR with the sequencing results of the fusion transcript in the three samples. The PCR primer is marked in panel A. D: The prediction of ORF of and its function for PTGFRN-NOTCH2 by bioinformatics, The start codon was using the start codon of NOTCH2 and the stop codon (red “*”) located in the fusion sequence from PTGFRN. The fusion peptide contained the domains (predicted by CD-Search in NCBI) in its region from NOTCH2, like EGF domains, but some key domains in the protein NOTCH2, such as NOTCH domain and Ankyrin repeats, were missing.

We used two algorithms, deFuse and TopHat-Fusion, to detect gene fusion based on the pair-ends reads in different samples. Although various results were generated by deFuse and TopHat-Fusion (Table S5), a fusion event between PTGFRN and NOTCH2 was the only cancer-specific fusion event identified by both algorithms. As shown in Fig. 6A, PTGFRN and NOTCH2 are separated on chromosome 1 by 3 million bps, and wild type forms are transcribed from opposite directions. In our cancer sample, we detected that the first intron of PTGFRN is fused with the 3′ junction of the 17th exon of NOTCH2 to generate a chimeric PTGFRN-NOTCH2 transcript. It is worth noting that a partial intronic region of PTGFRN is present in mature mRNA due to this fusion event (Fig. 6B). We thus separately designed a pair of primers that coordinate with the first intron of PTGFRN and the exon region of NOTCH2 to confirm this fusion in normal, adjacent non-tumor and cancer tissue by RT-PCR. The results indicated that this fusion event is cancer-restricted (Fig. 6C), which is consistent with the conclusions from our RNA-seq analysis. In addition, we examined the PTGFRN-NOTCH2 gene fusion in additional ten samples by RT-PCR, but none of them showed the gene fusion, suggesting that the PTGFRN-NOTCH2 might be a rare gene fusion in colorectal cancer.

Using strands-specific reverse-transcription and PCR, we found that the fusion gene transcribed with the promoter of NOTCH2. We predicted the ORF of fusion gene by using the start codon of NOTCH2 (Fig. 6D). This predicted protein corresponds to a 934aa peptide sequence with the first 917aa from NOTCH2 (these sequences were listed in Supplementary S1). We annotated the protein sequence using CD-Search tools in NCBI (http://www.ncbi.nlm.nih.gov/Structure/cdd/wrpsb.cgi, database: CDD), and found some EGF domains in the peptide regions from NOTCH2. However, some essential domains in NOTCH2, such as NOTCH domain and Ankyrin repeats, were lost in the fusion protein (Fig. 6D). Therefore, we inferred that the gene fusion PTGFRN-NOTCH2 in this study appeared to be more like a loss-of-function mutations, consistent with those recently described for myeloid leukemia [46], head and neck squamous cell carcinoma [47], [48]. Nevertheless, the overall expression of wild-type PTGFRN, NOTCH2 and its targets (PTCRA, HES1, HES5) are less affected in cancer (Table S6), indicating that 1) the PTGFRN-NOTCH2 fusion could occur in a subset of cancer cells or 2) that the fusion is heterozygous in cancer tissue and the fusion allele might be expressed at an extremely lower level. Further investigations are needed to understand the particular mechanism of this fusion event and its functional consequence.

## Discussion

Using RNA-seq technology, we profiled the whole transcriptomes from CRC, adjacent non-tumor and normal tissue with extremely thoroughly. In total, approximately 50–70 million reads were generated per sample, which enabled us to quantify the gene expression abundance at a wide range [49]. The number of expressed genes (FPKM >0) detected in our study is approximately 67% of the total UCSC reference genes per sample, representing the majority of the transcriptome.

Alternative regulation of gene expression can be achieved by transcriptional and post-transcriptional regulation. The first class of dysregulation of CRC at the transcriptional level has been well studied using microarray technology [14], [15], [50]. Quantifying the second class of regulatory change remains challenging despite the invention of the exon array [51]. RNA-seq technology enables the simultaneous study of these two different mechanisms [19], [26], [52], [53]. In our study, we investigated transcriptional dysregulation by analyzing the DEGs. Then, we used pair-end cDNA sequencing to more efficiently identify the alternative splicing. Moreover, by employing the MISO algorithm, we were able to measure the relative expression level of different isoforms produced by the exon-skipping events, which are quantitative measurements of alternative splicing. Interestingly, the genes affected by these two different regulatory mechanisms are largely independent (Fig. 4B), suggesting versatile ways to reprogram the cancer transcriptome.

The local invasion and distant metastasis of cancer has been considered a multistep processes composed of the regulatory changing of intracellular circuitry and the complex interaction between cancer cells and their microenvironment [36], [54], [55]. During invasion and metastasis, frequent remodeling of the extracellular matrix enables cancer cells to disseminate from primary tumors and invade normal tissue. In our study, we found that many genes related to extracellular matrix (ECM) receptor interactions are highly dysregulated in a cancer-restricted manner. The ECM is composed of several types of macromolecules, including collagen-type proteins, laminins, tenascin and other adhesion molecules [55]. All of the collagen-type genes, including type I–IX collagen, are up-regulated 10- to 1000-fold in cancer tissue (Table S7). Although there is some concordance between our observations and previous studies on the up-regulation of collagen mRNA in colorectal cancer tissue [56], the pervasive induction of collagen mRNAs is unique to our study. These findings suggest that the reprogramming of the collagen protein family network during colon cancer development can be much more complex than previously thought. In addition, we also noted that members of the matrix metalloproteinase (MMP) family, which degrade ECM structures [55], [57], are also significantly induced in cancer tissues, consistent with a previous report [58]. The fold change in the expression of the MMPs ranged from 10-fold (MMP1, MMP3 and MMP14) to 554-fold (MMP7). Meanwhile, other cell-cell adhesion-related molecules, such as laminins (LAMA4, LAMA5, LAMB1, LAMB2 and LAMC2) and integrins (ITGA5, ITGA5, ITGB5, ITGA11 and ITGBL1), are elevated in cancer tissues. We also detected the up-regulation of vascular endothelial growth factor (VEGF), suggesting that the “angiogenesis switch” is activated in cancer tissue. Taken together, the global up-regulation of the ECM pathway and the angiogenic growth factor indicates that CRC progression leads to massive ECM remodeling and the expansion of new vessel networks. Moreover, previous studies have shown that genes in the ECM pathway are under intensive epigenetic modification [59] and thus may be novel prognostic biomarkers; thus, our study provides greater insight into using expression changes in ECM pathway members as candidate biomarkers.

Gene fusion, which often results from a genomic aberration, has been shown to be the key mechanism for generating chimeric “oncogenes” that initiate tumorigenesis or contribute to tumor progression (reviewed in [60]). Using the RNA-seq technique, the expressed gene fusion transcript that is more likely to produce a functional product can be detected [23], [61]. Given that common gene fusion is rare in CRC [5], identifying case-specific gene fusion can help to understand the complexity of the molecular basis of CRC development. In this study, we detected a cancer-restricted gene fusion between PTGFRN and NOTCH2 in CRC. In addition, the gene fusions between the immunoglobulin lambda variables and IGLL5 were detected in the filtering result of TopHat-Fusion (Table S5), which might represent immune rearrangements in tumor-associated B cells. Previous studies suggested that the consequence of gene fusion can be i) an alteration of gene expression [62]; or ii) the generation of a truncated or chimeric protein with a different function [63]. Because the PTGFRN-NOTCH2 transcript only includes a small portion of PTGFRN and the expression of PTGFRN and NOTCH2 are not down-regulated in CRC, we reason that the original functions of these two genes are not affected by this fusion event, and therefore, the gain of function of this fusion construct will be particularly interesting for future study. Given that the majority of the fusion gene is composed of NOTCH2, the function of this fusion product could be more related to that for NOTCH2. NOTCH2 is a homolog of NOTCH1 and plays a role in a variety of developmental processes by controlling cell fate decisions. NOTCH2 expression has been shown to be a prognostic predictor and is related to the tumor differentiation status in CRC [64], [65]. In addition, the gain of function of truncated NOTCH2 with nonsense mutations causes an autosomal dominant skeletal disorder [66]. Therefore, NOTCH2 may play an important role in CRC development, and the PTGFRN-NOTCH2 gene fusion could introduce dominant negative effects on the normal development program.

## Materials and Methods

### Sample information

Written informed consent from the patients was obtained, and this series of studies was reviewed and approved by Institutional Ethics Committees of Fujian Provincial Hospital (Fuzhou, China). Three samples used in RNA-Seq, including distant normal colonic mucosa, adjacent colonic mucosa and cancer, were collected from one Chinese patient who was diagnosed with stage III colon adenocarcinoma. The distance between adjacent non-tumor and cancer tissue boundary is about 1cm, while that of distant normal tissue and cancer tissue is about 10 cm. Fig. S1 provides the micrograph of the cancer sample used in our study. Ten paired normal and cancer samples used in additional validation were obtained from ten patients with stage III colon adenocarcinoma.

### Library preparation

Total RNA was extracted from normal, adjacent non-tumor and cancerous colon tissues with TRIzol according to the manufacturer's protocol (Invitrogen). For mRNA-seq sample preparation, the Illumina standard kit was used according to the TruSeq RNA SamplePrep Guide (Illumina). Briefly, 10 µg of total RNA from each sample was used for polyA mRNA selection using poly T oligo-conjugated magnetic beads by two rounds of purification, followed by thermal mRNA fragmentation. The fragmented mRNA was subjected to cDNA synthesis using reverse transcriptase (SuperScript II) and random primers. The cDNA was further converted into double-stranded cDNA, and after end repair (Klenow fragment, T4 polynucleotide kinase, T4 polymerase and 3-‘A’ add process [Klenow exo-fragment]), the product was ligated to Illumina Truseq adaptors. Size selection was performed using a 2% agarose gel, generating 380-bp cDNA libraries. Finally, the libraries were enriched using 15 cycles of PCR and purified with the QIAquick PCR purification kit (Qiagen). The enriched libraries were diluted with elution buffer to a final concentration of 10 nM.

### Sequencing and primary analysis

Libraries from normal tissue, adjacent non-tumor tissue and cancerous colon tissue were analyzed at a concentration of 11 pM on a single Genome Analyzer *IIx* (GA*IIx*) lane using 115-bp sequencing. Raw RNA-seq data were filtered by Fastx-tools (http://hannonlab.cshl.edu/fastx_toolkit/) according to the following criteria: 1) reads containing sequencing adaptors were removed; 2) nucleotides with a quality score lower than 20 were trimmed from the end of the sequence; 3) reads shorter than 50 were discarded; and 4) artificial reads were removed. After the filtering pipeline, a total of 21.5G bp of cleaned, paired-end reads were produced. The raw sequence data have been submitted to the NCBI Short Read Archive with accession number SRP009386.

### RNA-seq reads mapping

The clean reads were then aligned with the UCSC *H. sapiens* reference genome (build hg19) using TopHat v1.3.1 [53], which initially removes a portion of the reads based on quality information accompanying each read and then maps the reads to the reference genome. The pre-built *H. sapiens* UCSC hg19 index was downloaded from the TopHat homepage and used as the reference genome. TopHat allows multiple alignments per read (up to 20 by default) and a maximum of two mismatches when mapping the reads to the reference. TopHat builds a database of potential splice junctions and confirms these by comparing the previously unmapped reads against the database of putative junctions. The default parameters for the TopHat method were used.

### Transcript abundance estimation

The aligned read files were processed by Cufflinks v1.0.3 [26], which uses the normalized RNA-seq fragment counts to measure the relative abundances of the transcripts. The unit of measurement is Fragments Per Kilobase of exon per Million fragments mapped (FPKM). Confidence intervals for FPKM estimates were calculated using a Bayesian inference method [67]. The reference GTF annotation file used in Cufflinks was downloaded from the Ensembl database (Homo_sapiens.GRCh37.63.gtf [68]). The transcript abundance data has been submitted to the GEO database with accession ID GSE33782.

### Differentially expressed gene testing

The downloaded Ensembl GTF file was passed to Cuffdiff along with the original alignment (SAM) files produced by TopHat. Cuffdiff re-estimates the abundance of the transcripts listed in the GTF file using alignments from the SAM file and concurrently tests for differential expression. Only the comparisons with “q_value” less than 0.01 and test status marked as “OK” in the Cuffidff output were regarded as showing differential expression.

### Detection of differential exon skipping events using MISO

The Mixture of Isoforms (MISO) analysis [42] was used to detect differentially regulated exons across samples. The MISO analysis was performed according to the tool's given workflow using paired-end reads (http://genes.mit.edu/burgelab/miso/docs/). The reads alignment files (SAM) produced by TopHat and the pre-build human genome (Hg19) alternative events downloaded from the MISO reference manual page (http://genes.mit.edu/burgelab/miso/docs/#gff-event-annotation) were used as the input.

### Filtering for highly reliable cancer-associated differential exon skipping **(**DES**)** events

To identify highly reliable cancer-associated DES events, we filtered the DES events by the flowing steps: 1) use the stringent cuff-offs to filter the result of MISO (the absolute value of diff >0.3 and bayes factor >1000, the default cut-off of MISO were 0.2 and 10); 2) remove the DES events occurred only in adjacent non-tumor tissue vs. normal tissue but not in CRC tissue vs. adjacent non-tumor and CRC tissue vs. normal tissue to ensure the events associated with the CRC tissue; 3) keep the genes that are overlapped with the cancer-associated gene set, which were collected from the NCBI gene database (searched by “oncogene” and “tumor suppressor gene”) and the Bushman Lab web (http://microb230.med.upenn.edu/protocols/cancergenes.html).

### Mapped reads visualization

The mapping results were visualized using the Integrative Genomics Viewer (IGV) available at http://www.broadinstitute.org/igv/. Views of other individual genes were generated by uploading coverage.wig files to the UCSC Genome browser.

### Functional enrichment analysis of differentially expressed genes

The Database for Annotation, Visualization and Integrated Discovery (DAVID) v6.7 is a set of web-based functional annotation tools [35]. The unique lists of differentially expressed genes and all the expressed genes (FPKM >0 in any sample) were submitted to the web interface as the gene list and background, respectively. The cut-off of the False Discovery Rate (FDR) was set at 5%, and only the results from the GO FAT and KEGG pathways were selected as functional annotation categories for this analysis.

### Candidate gene fusion identification

All the filtered RNA-seq reads were mapped to the reference transcript sequences that were downloaded from the Ensembl database (Homo_sapiens.GRCh37.63.cdna.all.fa) using TopHat. The read pairs mapping to the same transcripts were removed, and the ends of remaining reads were truncated to maintain the 75-bp length using in-house Perl scripts. These fixed-length reads were passed to two software packages, deFuse (deFuse-0.4.2) [61] and TopHat-Fusion (TopHatFusion-0.1.0) [69], to find the candidate gene fusions. The bowtie-index used in the TopHat-Fusion was downloaded from the TopHat homepage (H. sapiens UCSC hg19). The parameters of the TopHat-Fusion used were obtained from the “Getting Started” (http://tophat-fusion.sourceforge.net/tutorial.html) tutorial. The deFuse parameters were the default settings, as described in the deFuse manual.

### Candidate gene fusion filtering

The deFuse results were filtered according to McPherson et al. [61] and Steidl et al. [70]. There were 82 candidate gene fusions remaining after the filtering pipeline. The TopHat-Fusion results were parsed by in-house Perl scripts and filtered according to the following pipeline: 1) the span reads were greater than eight reads; 2) the ratio of the against reads vs. the span reads was less than 0.5; and 3) gene fusions involving ribosomal proteins or small nuclear ribosomal proteins were excluded. There were 11 filtered candidate fusions remaining after parsing. The filtering candidates simultaneously detected by both deFuse and TopHat-Fusion were regarded as reliable candidate gene fusions. After this filtering pipeline, one reliable candidate, PTGFRN-NOTCH2, was obtained and validated.

### Differentially expressed gene validation

The differentially expressed genes were validated by Real-Time Quantitative Polymerase Chain Reaction (RT-qPCR) using a LightCycler® 480 Instrument II (Roche). The PCR volume included 10 µl sample, 5 µl 2× SYBR Green Master Mix (TOYOBO), 1 µl cDNA template and 1 pmol/µl of each oligonucleotide. The RT-qPCR thermal profile was obtained using the following procedure: 95°C for 1 min, 40 cycles of 95°C for 10 sec, 60°C for 30 sec and 72°C for 10 sec, followed by 72°C for 5 min. The program was set to reveal the melting curve of each amplicon from 60°C to 95°C and obtain a read every 0.5°C. The primer sequences are listed in Table S2. All the RT-qPCR reactions were performed in triplicate to capture intra-assay variability.

The expression levels of each target gene in the tested experimental conditions (adjacent non-tumor and cancerous colon tissue) were compared to the control condition (normal colon tissue) according to Cook et al. [71]. The data were normalized using GAPDH, which had previously been identified as the best reference gene under different experimental conditions [72]. In the present analysis, GAPDH was confirmed to be stable and always showed variability less than ±1 cycle.

### Gene fusion validation

To detect fusion transcripts, we design the forward primer targeting the 5′ partner gene and reverse primer targeting the 3′ partner. Primer pairs (Table S2, NOTCH2 and PTGFRN) for the coding exons of the fusion genes were generated using Primer 5 software (PREMIER Biosoft International, Palo Alto, Calif.), and the PCR volume used comprised 10 µl sample, 1 µl 10× PCR buffer, 1 µl cDNA template, 0.2 µl dNTP, 0.2 µl Taq Enzyme (Genscript), and 0.2 pmol/µl each oligonucleotide. PCR was performed using the following procedure: 95°C for 1 min, 40 cycles of 95°C for 15 sec, 55°C for 30 sec and 72°C for 15 sec, followed by 72°C for 5 min. We confirmed the presence of the fusion gene in cancerous colon tissue. GAPDH was used as the loading control. The PCR products of the fusion gene were cloned in the pGEM®-T Easy Vector (Promega) and then sequenced with the T7 primer using a 3730 DNA Analyzer (ABI).

### Detecting the transcription direction of gene fusion

Total RNA (2 μg) was reverse transcribed into single-stranded cDNAs using SuperScript III reverse transcriptase (Invitrogen) and gene-special primer (Table S2, NOTCH2 and PTGFRN) in 20 μl reaction at 42°C for 60 min, 70°C for 15 min, 4°C for 5 min, respectively. 2 μl of cDNA was used for a subsequent 20 μl PCR amplification. To detect fusion transcripts, we design another primer pairs (Table S2, NOTCH2-nest and PTGFRN-nest) for the coding exons of the fusion genes were generated using Primer5 software. PCR was performed using the following procedure: 95°C for 1 min, 40 cycles of 95°C for 15 sec, 50°C for 20 sec and 72°C for 15 sec, followed by 72°C for 5 min. We confirmed the presence of the fusion gene in single-stranded cDNAs which were reverse-transcribed by PTGFRN-nest primer.

## Supporting Information

Figure S1 **Histological image of a hematoxylin/eosin-stained section of the colon cancer sample** (**original magnification ×100**).
(TIF)

Figure S2 **Pair-wise MA plots for all expressed genes among samples.** Each dots stands for one gene in comparison, the dotted line in grey indicates M = 0. Differentially expressed genes were plotted in red (up-regulated) and blue (down-regulated).
(TIF)

Figure S3 **Hierarchical clustering of the cancer and normal samples from nine patients based on five gene expression by qRT-PCR.** p1c indicates the cancer sample of patient 1, p1n indicates the normal sample of patient 1, and so on. The gene expression was measured as −ΔCT (ΔCT means the average of cycle number difference between the target gene and the control) in qRT-PCR and normalized by row. The cancer samples of patient 1, 3, 4, 5 and 6 (p1c, p3c – p6c) were clustered together.
(TIF)

Figure S4 **RNA-Seq reads mapping of exon skipping events for CTNND1.** The RNA-Seq reads were mapping to the UCSC reference genome (hg19) of CTNND1. The CRC tissue tracks are shown in red, the adjacent non-tumor in green and the normal tissue in blue. The distribution of MISO Ψ was shown in the right.
(TIF)

Figure S5 **RNA-Seq reads mapping of exon skipping events for PDGFA.** The RNA-Seq reads were mapping to the UCSC reference genome (hg19) of PDGFA. The CRC tissue tracks are shown in red, the adjacent non-tumor in green and the normal tissue in blue.
(TIF)

Figure S6 **RNA-Seq reads mapping of exon skipping events for EPB41L3.** The RNA-Seq reads were mapping to the UCSC reference genome (hg19) of EPB41L3. The CRC tissue tracks are shown in red, the adjacent non-tumor in green and the normal tissue in blue.
(TIF)

Figure S7 **RNA-Seq reads mapping of exon skipping events for F3.** The RNA-Seq reads were mapping to the UCSC reference genome (hg19) of F3. The CRC tissue tracks are shown in red, the adjacent non-tumor in green and the normal tissue in blue.
(TIF)

Figure S8 **RNA-Seq reads mapping of exon skipping events for MUC4.** The RNA-Seq reads were mapping to the UCSC reference genome (hg19) of MUC4. The CRC tissue tracks are shown in red, the adjacent non-tumor in green and the normal tissue in blue.
(TIF)

Table S1 **Gene expression and differentially expressed genes.**
(XLS)

Table S2 **The primer sequences used in qPCR and gene fusion validation.**
(XLS)

Table S3 **Fold change of gene expression** (**cancer/normal**) **for five ECM-pathway genes.**
(XLS)

Table S4 **Differential exon skipping events between normal, adjacent non-tumor and cancer tissues.**
(XLS)

Table S5 **Gene fusion analysis.**
(XLS)

Table S6 **Gene expression of PTGFRN, NOTCH gene and Notch-targed genes based on Notch signaling pathway in KEGG.**
(XLS)

Table S7 **Expression of collagen, MMP, laminins and intergrins.**
(XLS)

Supplementary S1 **Fusion sequence of PTGFRN-NOTCH2.**
(DOC)
