# Supplementary figures and images for: Transcriptome Profiling of the Cancer, Adjacent Non-Tumor and Distant Normal Tissues from a Colorectal Cancer Patient by Deep Sequencing
Source: PLoS One. 2012 Aug 8;7(8):e41001. doi: 10.1371/journal.pone.0041001 (PMC3414479; doi:10.1371/journal.pone.0041001)

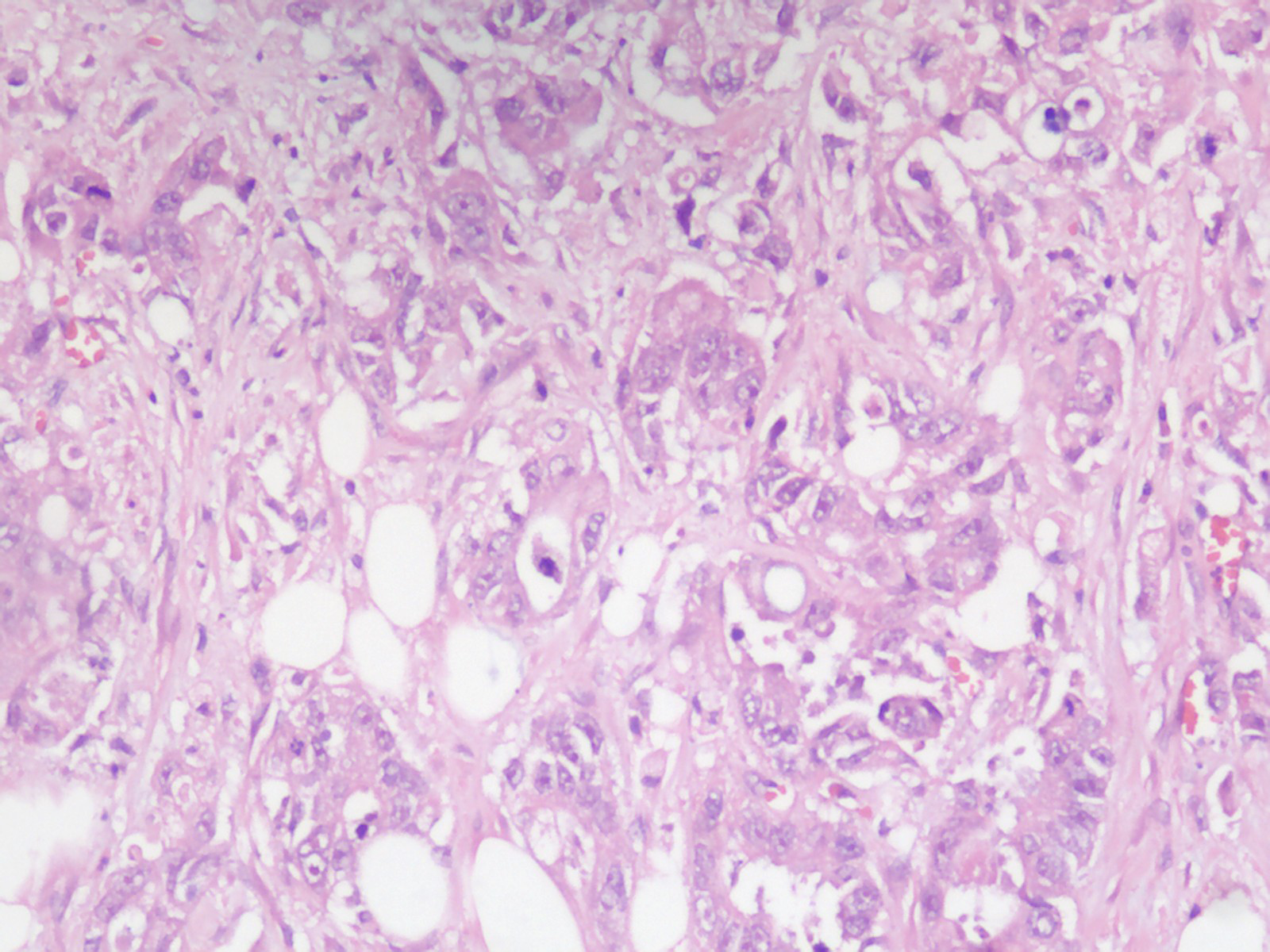

Supplement: Figure S1 — Histological image of a hematoxylin/eosin-stained section of the colon cancer sample (original magnification ×100). (TIF) [file pone.0041001.s001.tif]

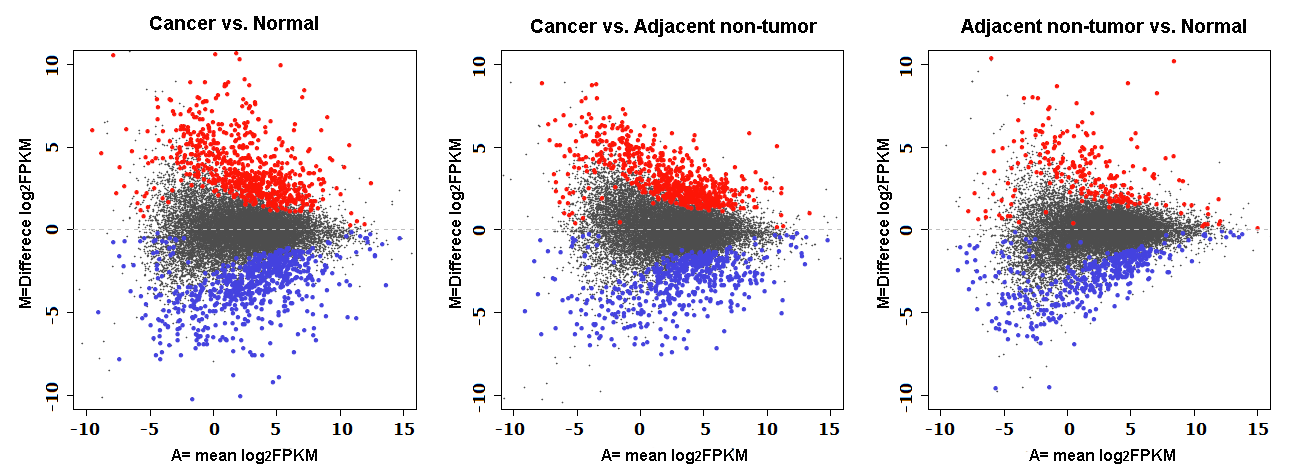

Supplement: Figure S2 — Pair-wise MA plots for all expressed genes among samples. Each dots stands for one gene in comparison, the dotted line in grey indicates M = 0. Differentially expressed genes were plotted in red (up-regulated) and blue (down-regulated). (TIF) [file pone.0041001.s002.tif]

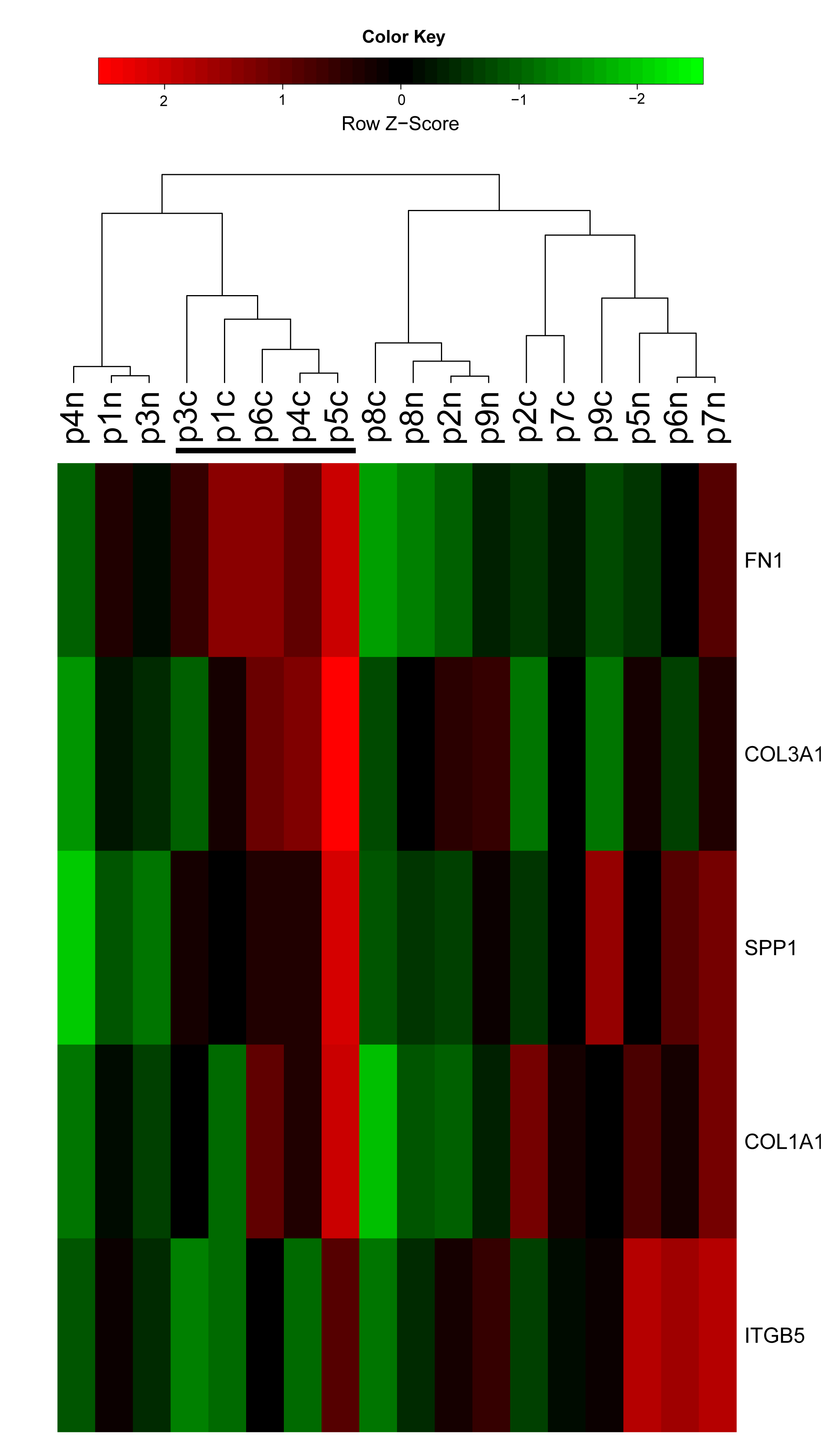

Supplement: Figure S3 — Hierarchical clustering of the cancer and normal samples from nine patients based on five gene expression by qRT-PCR. p1c indicates the cancer sample of patient 1, p1n indicates the normal sample of patient 1, and so on. The gene expression was measured as −ΔCT (ΔCT means the average of cycle number difference between the target gene and the control) in qRT-PCR and normalized by row. The cancer samples of patient 1, 3, 4, 5 and 6 (p1c, p3c – p6c) were clustered together. (TIF) [file pone.0041001.s003.tif]

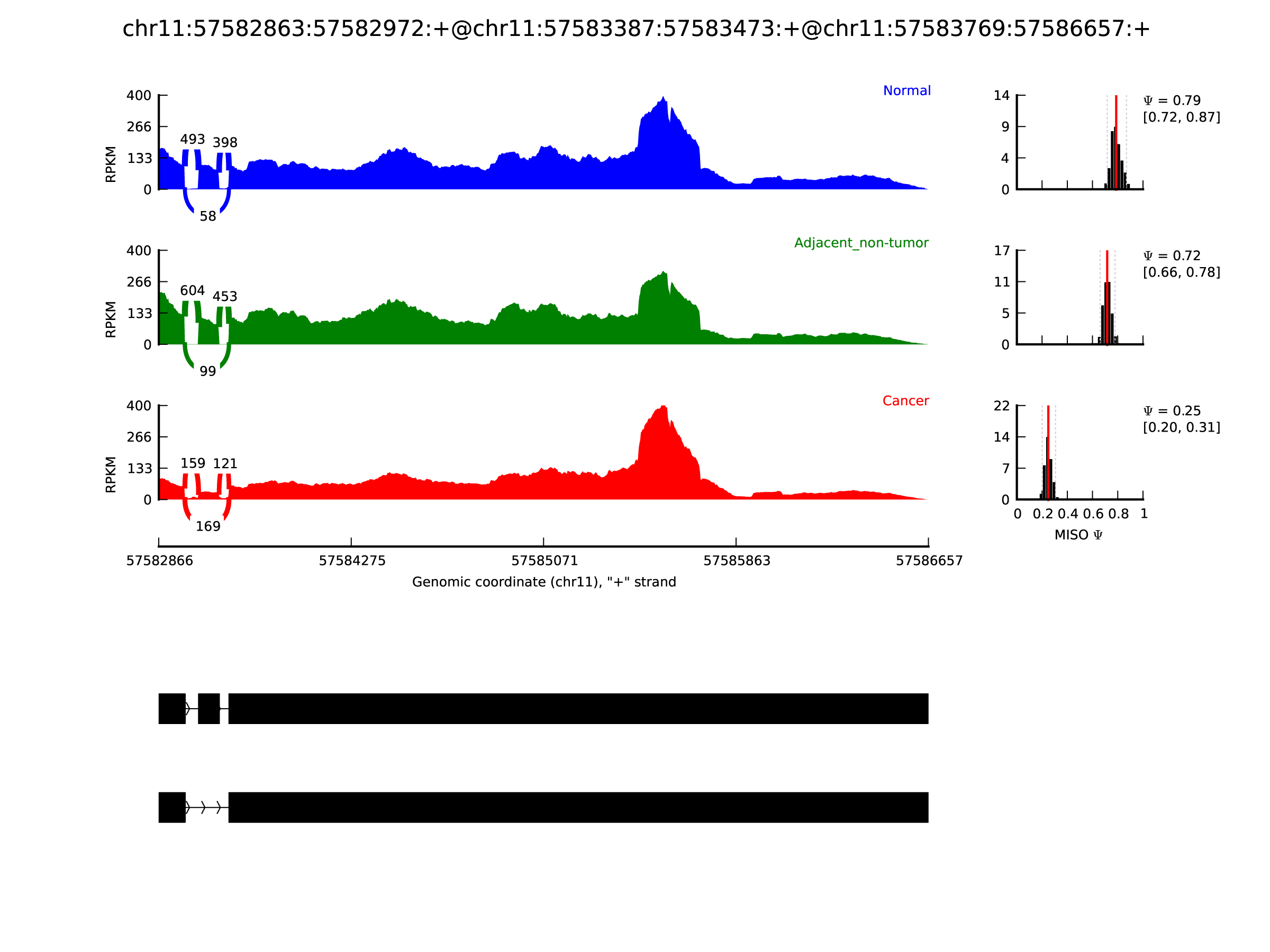

Supplement: Figure S4 — RNA-Seq reads mapping of exon skipping events for CTNND1. The RNA-Seq reads were mapping to the UCSC reference genome (hg19) of CTNND1. The CRC tissue tracks are shown in red, the adjacent non-tumor in green and the normal tissue in blue. The distribution of MISO Ψ was shown in the right. (TIF) [file pone.0041001.s004.tif]

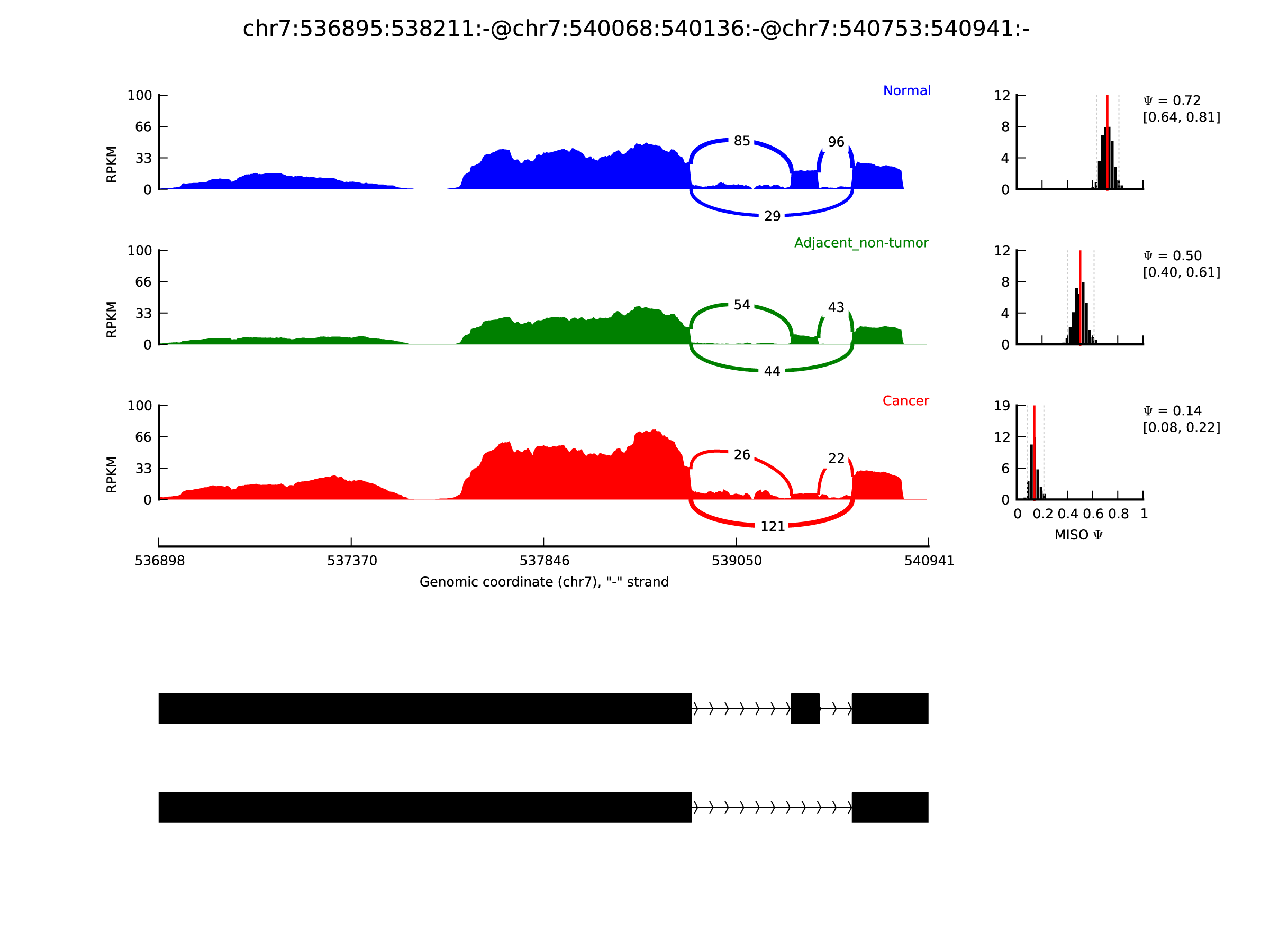

Supplement: Figure S5 — RNA-Seq reads mapping of exon skipping events for PDGFA. The RNA-Seq reads were mapping to the UCSC reference genome (hg19) of PDGFA. The CRC tissue tracks are shown in red, the adjacent non-tumor in green and the normal tissue in blue. (TIF) [file pone.0041001.s005.tif]

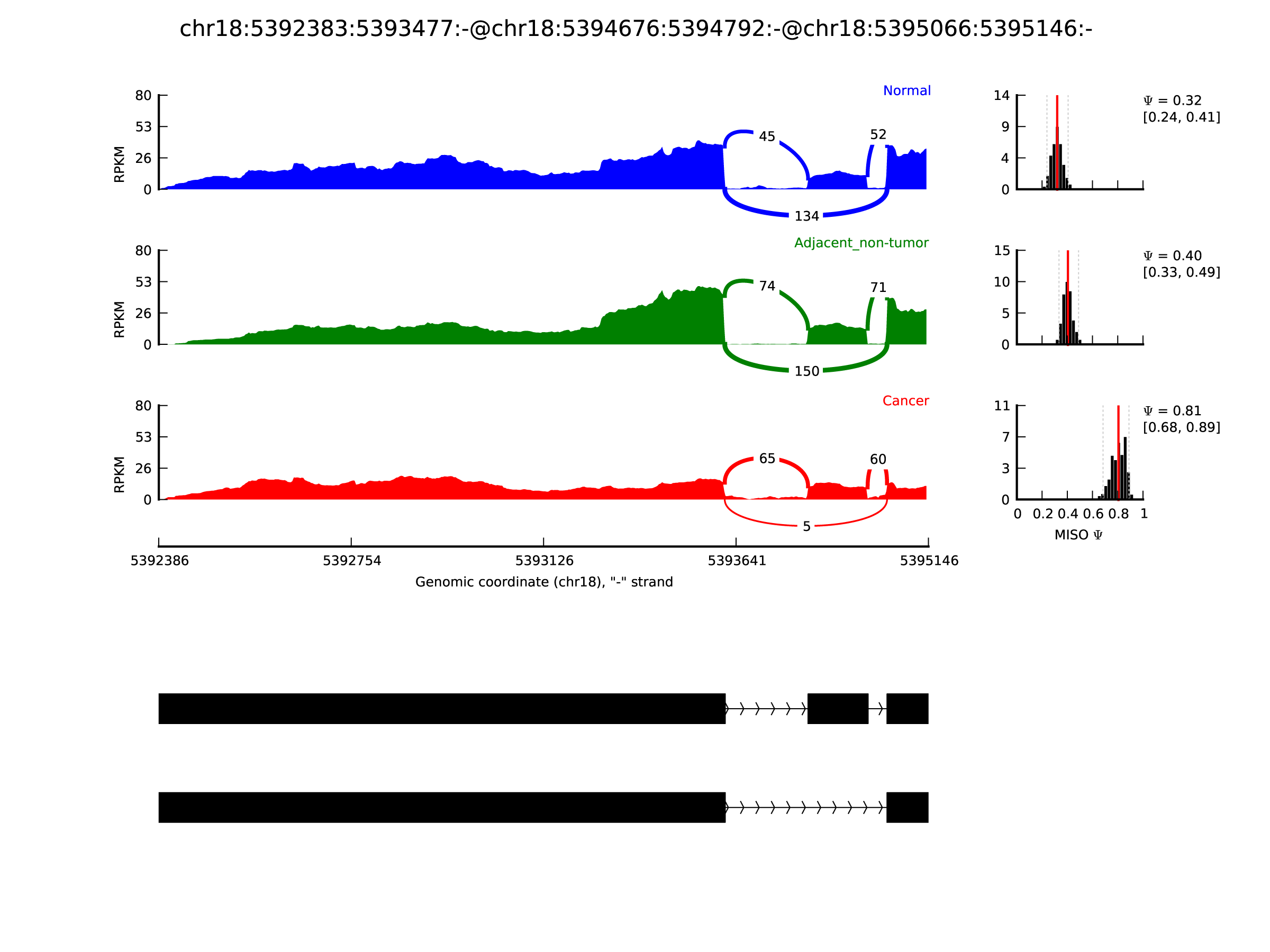

Supplement: Figure S6 — RNA-Seq reads mapping of exon skipping events for EPB41L3. The RNA-Seq reads were mapping to the UCSC reference genome (hg19) of EPB41L3. The CRC tissue tracks are shown in red, the adjacent non-tumor in green and the normal tissue in blue. (TIF) [file pone.0041001.s006.tif]

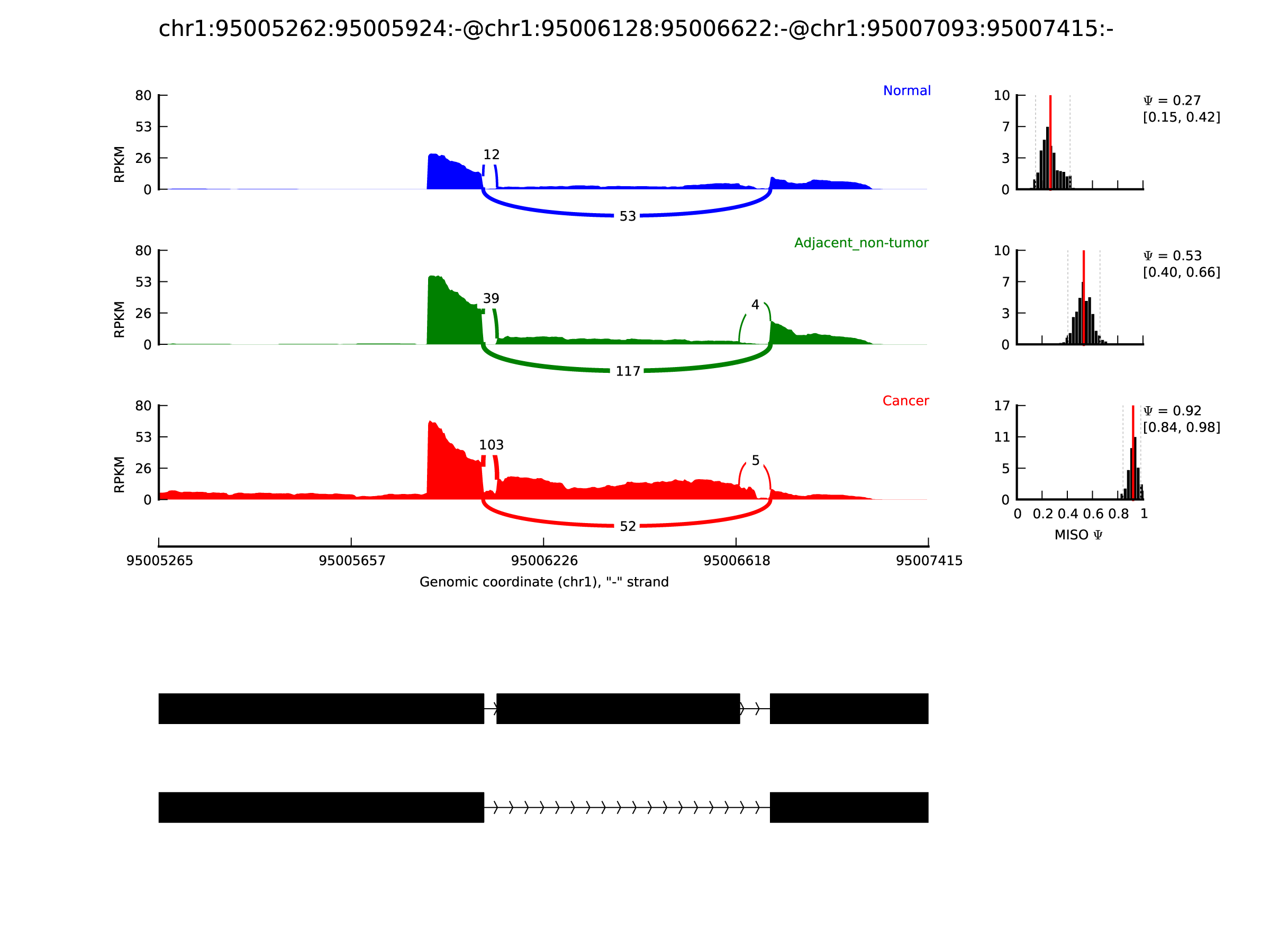

Supplement: Figure S7 — RNA-Seq reads mapping of exon skipping events for F3. The RNA-Seq reads were mapping to the UCSC reference genome (hg19) of F3. The CRC tissue tracks are shown in red, the adjacent non-tumor in green and the normal tissue in blue. (TIF) [file pone.0041001.s007.tif]

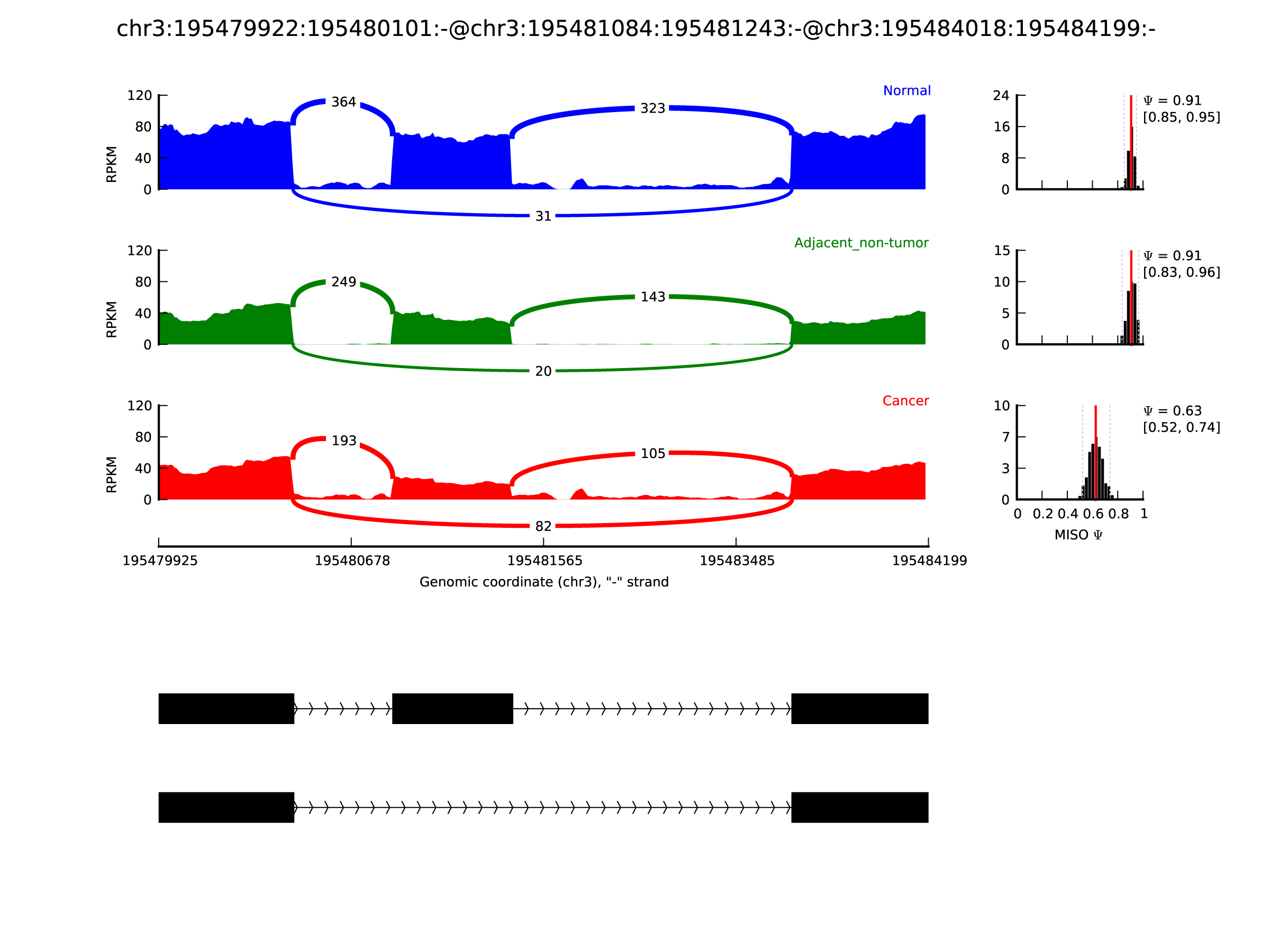

Supplement: Figure S8 — RNA-Seq reads mapping of exon skipping events for MUC4. The RNA-Seq reads were mapping to the UCSC reference genome (hg19) of MUC4. The CRC tissue tracks are shown in red, the adjacent non-tumor in green and the normal tissue in blue. (TIF) [file pone.0041001.s008.tif]
