# Supplementary material for: Transcriptome Profiling of the Cancer, Adjacent Non-Tumor and Distant Normal Tissues from a Colorectal Cancer Patient by Deep Sequencing
Source: PLoS One. 2012 Aug 8;7(8):e41001. doi: 10.1371/journal.pone.0041001 (PMC3414479; doi:10.1371/journal.pone.0041001)
Supplement: Supplementary S1 — Fusion sequence of PTGFRN-NOTCH2. (DOC) [file pone.0041001.s016.doc]

## Fusion sequence of PTGFRN-NOTCH2

Note: the capital bases of following sequence are from NOTCH2, the lowercase are from PTGFRN.

- The fusion sequence of gene fusion PTGFRN-NOTCH2 provided by deFuse.

>PTGFRN-NOTCH2 fusion sequence 514bp

AGACAGTATTGGCTCCCTGTTCCCCAAACCCTTGTGAGAATGCTGCTGTTTGCAAAGAGTCACCAAATTTTGAGAGTTATACTTGCTTGTGTGCTCCTGGCTGGCAAGGTCAGCGGTGTACCATTGACATTGACGAGTGTATCTCCAAGCCCTGCATGAACCATGGTCTCTGCCATAACACCCAGGGCAGCTACATGTGTGAATGTCCACCAGGCTTCAGTGGTATGGACTGTGAGGAGGACATTGATGACTGCCTTGCCAgggctggaatggtgatgacgactaccaaagtgatcttagaaactgtgagttgaagacagcagaactgccaccagctggggagccagaatgactgtgtaaagcagggttcccacccaaccctttcctctgaagcatctccctcgcattgttatataagacacatctattgtctgaaccactatgctttgagtttgttagagcagtttagcctacctgattaatacaaagtccttttatatcatttgaattttaa

- The predicted cDNA sequence of PTGFRN-NOTCH2.

>PTGFRN-NOTCH2 cDNA 3225bp

AAACTTCGGGCGGCGGCTGAGGCGGCGGCCGAGGAGCGGCGGACTCGGGGCGCGGGGAGTCGAGGCATTTGCGCCTGGGCTTCGGAGCGTAGCGCCAGGGCCTGAGCCTTTGAAGCAGGAGGAGGGGAGGAGAGAGTGGGGCTCCTCTATCGGGACCCCCTCCCCATGTGGATCTGCCCAGGCGGCGGCGGCGGCGGCGGAGGAGGAGGCGACCGAGAAGATGCCCGCCCTGCGCCCCGCTCTGCTGTGGGCGCTGCTGGCGCTCTGGCTGTGCTGCGCGGCCCCCGCGCATGCATTGCAGTGTCGAGATGGCTATGAACCCTGTGTAAATGAAGGAATGTGTGTTACCTACCACAATGGCACAGGATACTGCAAATGTCCAGAAGGCTTCTTGGGGGAATATTGTCAACATCGAGACCCCTGTGAGAAGAACCGCTGCCAGAATGGTGGGACTTGTGTGGCCCAGGCCATGCTGGGGAAAGCCACGTGCCGATGTGCCTCAGGGTTTACAGGAGAGGACTGCCAGTACTCAACATCTCATCCATGCTTTGTGTCTCGACCCTGCCTGAATGGCGGCACATGCCATATGCTCAGCCGGGATACCTATGAGTGCACCTGTCAAGTCGGGTTTACAGGTAAGGAGTGCCAATGGACGGATGCCTGCCTGTCTCATCCCTGTGCAAATGGAAGTACCTGTACCACTGTGGCCAACCAGTTCTCCTGCAAATGCCTCACAGGCTTCACAGGGCAGAAATGTGAGACTGATGTCAATGAGTGTGACATTCCAGGACACTGCCAGCATGGTGGCACCTGCCTCAACCTGCCTGGTTCCTACCAGTGCCAGTGCCCTCAGGGCTTCACAGGCCAGTACTGTGACAGCCTGTATGTGCCCTGTGCACCCTCACCTTGTGTCAATGGAGGCACCTGTCGGCAGACTGGTGACTTCACTTTTGAGTGCAACTGCCTTCCAGGTTTTGAAGGGAGCACCTGTGAGAGGAATATTGATGACTGCCCTAACCACAGGTGTCAGAATGGAGGGGTTTGTGTGGATGGGGTCAACACTTACAACTGCCGCTGTCCCCCACAATGGACAGGACAGTTCTGCACAGAGGATGTGGATGAATGCCTGCTGCAGCCCAATGCCTGTCAAAATGGGGGCACCTGTGCCAACCGCAATGGAGGCTATGGCTGTGTATGTGTCAACGGCTGGAGTGGAGATGACTGCAGTGAGAACATTGATGATTGTGCCTTCGCCTCCTGTACTCCAGGCTCCACCTGCATCGACCGTGTGGCCTCCTTCTCTTGCATGTGCCCAGAGGGGAAGGCAGGTCTCCTGTGTCATCTGGATGATGCATGCATCAGCAATCCTTGCCACAAGGGGGCACTGTGTGACACCAACCCCCTAAATGGGCAATATATTTGCACCTGCCCACAAGGCTACAAAGGGGCTGACTGCACAGAAGATGTGGATGAATGTGCCATGGCCAATAGCAATCCTTGTGAGCATGCAGGAAAATGTGTGAACACGGATGGCGCCTTCCACTGTGAGTGTCTGAAGGGTTATGCAGGACCTCGTTGTGAGATGGACATCAATGAGTGCCATTCAGACCCCTGCCAGAATGATGCTACCTGTCTGGATAAGATTGGAGGCTTCACATGTCTGTGCATGCCAGGTTTCAAAGGTGTGCATTGTGAATTAGAAATAAATGAATGTCAGAGCAACCCTTGTGTGAACAATGGGCAGTGTGTGGATAAAGTCAATCGTTTCCAGTGCCTGTGTCCTCCTGGTTTCACTGGGCCAGTTTGCCAGATTGATATTGATGACTGTTCCAGTACTCCGTGTCTGAATGGGGCAAAGTGTATCGATCACCCGAATGGCTATGAATGCCAGTGTGCCACAGGTTTCACTGGTGTGTTGTGTGAGGAGAACATTGACAACTGTGACCCCGATCCTTGCCACCATGGTCAGTGTCAGGATGGTATTGATTCCTACACCTGCATCTGCAATCCCGGGTACATGGGCGCCATCTGCAGTGACCAGATTGATGAATGTTACAGCAGCCCTTGCCTGAACGATGGTCGCTGCATTGACCTGGTCAATGGCTACCAGTGCAACTGCCAGCCAGGCACGTCAGGGGTTAATTGTGAAATTAATTTTGATGACTGTGCAAGTAACCCTTGTATCCATGGAATCTGTATGGATGGCATTAATCGCTACAGTTGTGTCTGCTCACCAGGATTCACAGGGCAGAGATGTAACATTGACATTGATGAGTGTGCCTCCAATCCCTGTCGCAAGGGTGCAACATGTATCAACGGTGTGAATGGTTTCCGCTGTATATGCCCCGAGGGACCCCATCACCCCAGCTGCTACTCACAGGTGAACGAATGCCTGAGCAATCCCTGCATCCATGGAAACTGTACTGGAGGTCTCAGTGGATATAAGTGTCTCTGTGATGCAGGCTGGGTTGGCATCAACTGTGAAGTGGACAAAAATGAATGCCTTTCGAATCCATGCCAGAATGGAGGAACTTGTGACAATCTGGTGAATGGATACAGGTGTACTTGCAAGAAGGGCTTTAAAGGCTATAACTGCCAGGTGAATATTGATGAATGTGCCTCAAATCCATGCCTGAACCAAGGAACCTGCTTTGATGACATAAGTGGCTACACTTGCCACTGTGTGCTGCCATACACAGGCAAGAATTGTCAGACAGTATTGGCTCCCTGTTCCCCAAACCCTTGTGAGAATGCTGCTGTTTGCAAAGAGTCACCAAATTTTGAGAGTTATACTTGCTTGTGTGCTCCTGGCTGGCAAGGTCAGCGGTGTACCATTGACATTGACGAGTGTATCTCCAAGCCCTGCATGAACCATGGTCTCTGCCATAACACCCAGGGCAGCTACATGTGTGAATGTCCACCAGGCTTCAGTGGTATGGACTGTGAGGAGGACATTGATGACTGCCTTGCCAgggctggaatggtgatgacgactaccaaagtgatcttagaaactgtgagttgaagacagcagaactgccaccagctggggagccagaatgactgtgtaaagcagggttcccacccaaccctttcctctgaagcatctccctcgcattgttatataagacacatctattgtctgaaccactatgctttgagtttgttagagcagtttagcctacctgattaatacaaagtccttttatatcatttgaattttaa

- The predicted ORF sequence of PTGFRN-NOTCH2.

>PTGFRN-NOTCH2 ORF 2805bp

ATGCCCGCCCTGCGCCCCGCTCTGCTGTGGGCGCTGCTGGCGCTCTGGCTGTGCTGCGCGGCCCCCGCGCATGCATTGCAGTGTCGAGATGGCTATGAACCCTGTGTAAATGAAGGAATGTGTGTTACCTACCACAATGGCACAGGATACTGCAAATGTCCAGAAGGCTTCTTGGGGGAATATTGTCAACATCGAGACCCCTGTGAGAAGAACCGCTGCCAGAATGGTGGGACTTGTGTGGCCCAGGCCATGCTGGGGAAAGCCACGTGCCGATGTGCCTCAGGGTTTACAGGAGAGGACTGCCAGTACTCAACATCTCATCCATGCTTTGTGTCTCGACCCTGCCTGAATGGCGGCACATGCCATATGCTCAGCCGGGATACCTATGAGTGCACCTGTCAAGTCGGGTTTACAGGTAAGGAGTGCCAATGGACGGATGCCTGCCTGTCTCATCCCTGTGCAAATGGAAGTACCTGTACCACTGTGGCCAACCAGTTCTCCTGCAAATGCCTCACAGGCTTCACAGGGCAGAAATGTGAGACTGATGTCAATGAGTGTGACATTCCAGGACACTGCCAGCATGGTGGCACCTGCCTCAACCTGCCTGGTTCCTACCAGTGCCAGTGCCCTCAGGGCTTCACAGGCCAGTACTGTGACAGCCTGTATGTGCCCTGTGCACCCTCACCTTGTGTCAATGGAGGCACCTGTCGGCAGACTGGTGACTTCACTTTTGAGTGCAACTGCCTTCCAGGTTTTGAAGGGAGCACCTGTGAGAGGAATATTGATGACTGCCCTAACCACAGGTGTCAGAATGGAGGGGTTTGTGTGGATGGGGTCAACACTTACAACTGCCGCTGTCCCCCACAATGGACAGGACAGTTCTGCACAGAGGATGTGGATGAATGCCTGCTGCAGCCCAATGCCTGTCAAAATGGGGGCACCTGTGCCAACCGCAATGGAGGCTATGGCTGTGTATGTGTCAACGGCTGGAGTGGAGATGACTGCAGTGAGAACATTGATGATTGTGCCTTCGCCTCCTGTACTCCAGGCTCCACCTGCATCGACCGTGTGGCCTCCTTCTCTTGCATGTGCCCAGAGGGGAAGGCAGGTCTCCTGTGTCATCTGGATGATGCATGCATCAGCAATCCTTGCCACAAGGGGGCACTGTGTGACACCAACCCCCTAAATGGGCAATATATTTGCACCTGCCCACAAGGCTACAAAGGGGCTGACTGCACAGAAGATGTGGATGAATGTGCCATGGCCAATAGCAATCCTTGTGAGCATGCAGGAAAATGTGTGAACACGGATGGCGCCTTCCACTGTGAGTGTCTGAAGGGTTATGCAGGACCTCGTTGTGAGATGGACATCAATGAGTGCCATTCAGACCCCTGCCAGAATGATGCTACCTGTCTGGATAAGATTGGAGGCTTCACATGTCTGTGCATGCCAGGTTTCAAAGGTGTGCATTGTGAATTAGAAATAAATGAATGTCAGAGCAACCCTTGTGTGAACAATGGGCAGTGTGTGGATAAAGTCAATCGTTTCCAGTGCCTGTGTCCTCCTGGTTTCACTGGGCCAGTTTGCCAGATTGATATTGATGACTGTTCCAGTACTCCGTGTCTGAATGGGGCAAAGTGTATCGATCACCCGAATGGCTATGAATGCCAGTGTGCCACAGGTTTCACTGGTGTGTTGTGTGAGGAGAACATTGACAACTGTGACCCCGATCCTTGCCACCATGGTCAGTGTCAGGATGGTATTGATTCCTACACCTGCATCTGCAATCCCGGGTACATGGGCGCCATCTGCAGTGACCAGATTGATGAATGTTACAGCAGCCCTTGCCTGAACGATGGTCGCTGCATTGACCTGGTCAATGGCTACCAGTGCAACTGCCAGCCAGGCACGTCAGGGGTTAATTGTGAAATTAATTTTGATGACTGTGCAAGTAACCCTTGTATCCATGGAATCTGTATGGATGGCATTAATCGCTACAGTTGTGTCTGCTCACCAGGATTCACAGGGCAGAGATGTAACATTGACATTGATGAGTGTGCCTCCAATCCCTGTCGCAAGGGTGCAACATGTATCAACGGTGTGAATGGTTTCCGCTGTATATGCCCCGAGGGACCCCATCACCCCAGCTGCTACTCACAGGTGAACGAATGCCTGAGCAATCCCTGCATCCATGGAAACTGTACTGGAGGTCTCAGTGGATATAAGTGTCTCTGTGATGCAGGCTGGGTTGGCATCAACTGTGAAGTGGACAAAAATGAATGCCTTTCGAATCCATGCCAGAATGGAGGAACTTGTGACAATCTGGTGAATGGATACAGGTGTACTTGCAAGAAGGGCTTTAAAGGCTATAACTGCCAGGTGAATATTGATGAATGTGCCTCAAATCCATGCCTGAACCAAGGAACCTGCTTTGATGACATAAGTGGCTACACTTGCCACTGTGTGCTGCCATACACAGGCAAGAATTGTCAGACAGTATTGGCTCCCTGTTCCCCAAACCCTTGTGAGAATGCTGCTGTTTGCAAAGAGTCACCAAATTTTGAGAGTTATACTTGCTTGTGTGCTCCTGGCTGGCAAGGTCAGCGGTGTACCATTGACATTGACGAGTGTATCTCCAAGCCCTGCATGAACCATGGTCTCTGCCATAACACCCAGGGCAGCTACATGTGTGAATGTCCACCAGGCTTCAGTGGTATGGACTGTGAGGAGGACATTGATGACTGCCTTGCCAgggctggaatggtgatgacgactaccaaagtgatcttagaaactgtgagttga

- The predicted peptide sequence of PTGFRN-NOTCH2.

>PTGFRN-NOTCH2 peptide 934aa

MPALRPALLWALLALWLCCAAPAHALQCRDGYEPCVNEGMCVTYHNGTGYCKCPEGFLGEYCQHRDPCEKNRCQNGGTCVAQAMLGKATCRCASGFTGEDCQYSTSHPCFVSRPCLNGGTCHMLSRDTYECTCQVGFTGKECQWTDACLSHPCANGSTCTTVANQFSCKCLTGFTGQKCETDVNECDIPGHCQHGGTCLNLPGSYQCQCPQGFTGQYCDSLYVPCAPSPCVNGGTCRQTGDFTFECNCLPGFEGSTCERNIDDCPNHRCQNGGVCVDGVNTYNCRCPPQWTGQFCTEDVDECLLQPNACQNGGTCANRNGGYGCVCVNGWSGDDCSENIDDCAFASCTPGSTCIDRVASFSCMCPEGKAGLLCHLDDACISNPCHKGALCDTNPLNGQYICTCPQGYKGADCTEDVDECAMANSNPCEHAGKCVNTDGAFHCECLKGYAGPRCEMDINECHSDPCQNDATCLDKIGGFTCLCMPGFKGVHCELEINECQSNPCVNNGQCVDKVNRFQCLCPPGFTGPVCQIDIDDCSSTPCLNGAKCIDHPNGYECQCATGFTGVLCEENIDNCDPDPCHHGQCQDGIDSYTCICNPGYMGAICSDQIDECYSSPCLNDGRCIDLVNGYQCNCQPGTSGVNCEINFDDCASNPCIHGICMDGINRYSCVCSPGFTGQRCNIDIDECASNPCRKGATCINGVNGFRCICPEGPHHPSCYSQVNECLSNPCIHGNCTGGLSGYKCLCDAGWVGINCEVDKNECLSNPCQNGGTCDNLVNGYRCTCKKGFKGYNCQVNIDECASNPCLNQGTCFDDISGYTCHCVLPYTGKNCQTVLAPCSPNPCENAAVCKESPNFESYTCLCAPGWQGQRCTIDIDECISKPCMNHGLCHNTQGSYMCECPPGFSGMDCEEDIDDCLAragmvmtttkviletvs*
